# SCL/TAL1 cooperates with Polycomb RYBP-PRC1 to suppress alternative lineages in blood-fated cells

**DOI:** 10.1038/s41467-018-07787-6

**Published:** 2018-12-18

**Authors:** Hedia Chagraoui, Maiken S. Kristiansen, Juan Pablo Ruiz, Ana Serra-Barros, Johanna Richter, Elisa Hall-Ponselé, Nicki Gray, Dominic Waithe, Kevin Clark, Philip Hublitz, Emmanouela Repapi, Georg Otto, Paul Sopp, Stephen Taylor, Supat Thongjuea, Paresh Vyas, Catherine Porcher

**Affiliations:** 1 Medical Research Council Molecular Haematology Unit, University of Oxford, John Radcliffe Hospital, Oxford, OX3 9DS UK; 2 Haematology Branch, National Heart, Lung and Blood Institute, National Institutes of Health, Bethesda, MD 20892 USA; 3 Computational Biology Research Group, University of Oxford, John Radcliffe Hospital, Oxford, OX3 9DS UK; 4 Wolfson Imaging Centre, University of Oxford, John Radcliffe Hospital, Oxford, OX3 9DS UK; 5 FACS Facility, University of Oxford, John Radcliffe Hospital, Oxford, OX3 9DS UK; 6 Genome Engineering Facility, Medical Research Council Weatherall Institute of Molecular Medicine, Radcliffe Department of Medicine, University of Oxford, John Radcliffe Hospital, Oxford, OX3 9DS UK; 7 Oxford National Institute for Health Research, Biomedical Research Centre, Haematology Theme, Oxford University Hospital, Oxford, OX3 9DU UK; 8 Present Address: Medimmune, Granta Park, CB21 6GH Cambridge, UK; 9 Present Address: MRC Centre for Regenerative Medicine, SCRM Building, The University of Edinburgh, Edinburgh, EH16 4UU UK; 10 Present Address: Genetics and Genomic Medicine, UCL Great Ormond Street Institute of Child Health, London, WC1N 1EH UK

## Abstract

During development, it is unclear if lineage-fated cells derive from multilineage-primed progenitors and whether active mechanisms operate to restrict cell fate. Here we investigate how mesoderm specifies into blood-fated cells. We document temporally restricted co-expression of blood (*Scl/Tal1*), cardiac (*Mesp1*) and paraxial (*Tbx6*) lineage-affiliated transcription factors in single cells, at the onset of blood specification, supporting the existence of common progenitors. At the same time-restricted stage, absence of SCL results in expansion of cardiac/paraxial cell populations and increased cardiac/paraxial gene expression, suggesting active suppression of alternative fates. Indeed, SCL normally activates expression of co-repressor ETO2 and Polycomb-PRC1 subunits (RYBP, PCGF5) and maintains levels of Polycomb-associated histone marks (H2AK119ub/H3K27me3). Genome-wide analyses reveal ETO2 and RYBP co-occupy most SCL target genes, including cardiac/paraxial loci. Reduction of *Eto2* or *Rybp* expression mimics *Scl*-null cardiac phenotype. Therefore, SCL-mediated transcriptional repression prevents mis-specification of blood-fated cells, establishing active repression as central to fate determination processes.

## Abstract (web summary)

Mechanisms that operate during embryonic development to restrict cell fate are currently under investigation. Here the authors characterise the role of SCL/TAL1 at the onset of blood specification in embryonic development using mouse EB differentiation culture as a model system.

## Introduction

In embryonic development, early cell fate decisions occur at gastrulation when epiblast cells migrate through the primitive streak (PS) and specify into germ layers^1–4^. A characteristic of cells undergoing specification is their developmental plasticity, as revealed by their ability to respond to changing environmental or intrinsic cues and adopt different fates, suggesting multipotency^3^. Cellular identity becomes locked after egression from the PS. Therefore, acquisition of lineage-specific features is likely to coincide with progressive loss of the ability to generate alternative lineages through restriction of cell potential. Whether emerging tissue-specific transcriptional regulators driving lineage specification also actively control these restriction processes is unclear.

Specification of blood cells from FLK1^+^ mesodermal progenitors is a model of lineage development. The initial steps of this process are under control of the transcription factor (TF) SCL/TAL1, placing it at the apex of the haematopoietic transcriptional hierarchy^5,6^. Interestingly, absence of SCL not only leads to complete block in haematopoiesis^7,8^, but also to expansion of the heart field in zebrafish embryos and ectopic cardiomyocyte production from mouse yolk sac and ES cell-derived endothelium, revealing latent cardiac potential in blood/endothelial progenitors^9–11^. Conversely, forced expression of *Scl* mRNA expands blood and endothelial tissues at the expense of myocardial tissues in vivo^12^ and in vitro^13^. This highlights a close developmental relationship between blood and cardiac lineages and supports the notion of plasticity.

However, it is unclear if common, multilineage-primed blood/cardiac mesodermal progenitors exist and whether active repression mechanisms are established in blood-fated cells to prevent development of the cardiac lineage. Two recent studies propose contrasting mechanisms. Molecular analyses of ES cell-derived FLK1^+^ cells show that SCL occupies a subset of enhancers regulating cardiac-specific genes, suggesting this makes these enhancers unavailable for activation by cardiac-specific TFs^11^. In contrast, single cell analyses from mouse *Scl*^*−/−*^ embryos failed to detect increased cardiac gene expression in *Scl*^*−/−*^ FLK1^+^ cells, questioning the role of SCL in suppressing the cardiac fate^14^. However, it is unclear if the two studies were conducted at similar developmental time points and examined functionally equivalent FLK1^+^ cells.

Mechanistically, SCL is both an activating and repressive TF. It acts within multi-protein complexes containing a core of four proteins (SCL/E47/LMO2/LDB1) and co-factors/chromatin remodelling proteins conferring activating (P300/CBP) or repressive (mSIN3A, ETO2, GFI1B) activities^5,15^.

Chromatin remodelling proteins, like repressive Polycomb (PcG) complexes, play critical functions in early development. PcG complexes control pluripotency and differentiation of embryonic stem (ES) cells and, in vivo, are required for survival and organogenesis^16^. Two PcG complexes (PRC1/PRC2) usually work in concert. Their activities are associated with distinct histone modifications: H2AK119 monoubiquitination (H2AK119ub, PRC1) and H3K27 trimethylation (H3K27me3, PRC2). Several PcG complexes exist that all contain enzymatic activities (PRC1 ubiquitin ligases; PRC2 methyltransferases), but vary in their overall composition. PRC1 complexes include ubiquitin ligase modules (RING1A/1B and PCGF1–6) and CBX or RYBP/YAF2 proteins in a mutually exclusive manner^17^. PcG complexes commonly bind CpG islands at gene promoters^18^.

To get further insight into the mechanisms underlying blood specification, we used murine ES cell differentiation cultures to follow production of mesoderm-derived blood-fated cells. We report a series of molecular events that occur over a restricted, one-day developmental time-window, at the onset of blood specification. We first document multi-lineage (blood/cardiac/paraxial) priming in single mesodermal cells. We then show that absence of SCL leads to rapid conversion of blood-fated cells into functional cardiac and paraxial cells, in agreement with the notion of cellular plasticity. To suppress alternative lineages, SCL activates expression of select repressors (ETO2 and PRC1 members) and creates a global repressive epigenetic environment, in parallel to activating blood/endothelial-related genes to promote haematopoietic specification. These processes form the basis of lineage selection and highlight the prevalence of active transcriptional repression in cell fate choices.

## Results

### Transient co-expression of distinct lineage-affiliated TFs

**Fig. 1:**
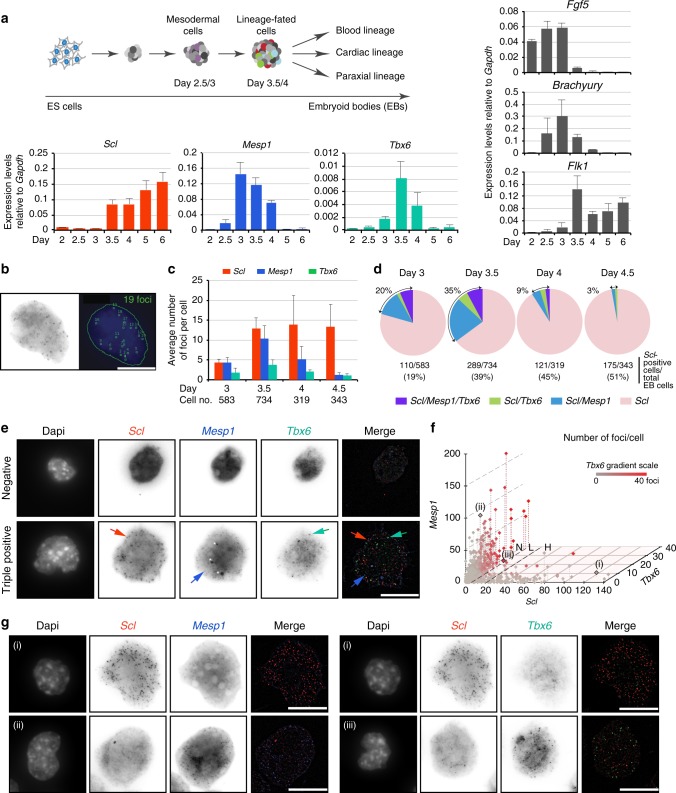
*Scl*, *Mesp1* and *Tbx6* are transiently co-expressed in single cells. **a** Top, Schematic of ES/EB in vitro differentiation. Right and bottom, RT-qPCR gene expression analyses from RNA isolated from day 2–6 EB cells (*n* = 3, mean ± SD). **b** Example of detection of single mRNA molecules (foci) in a single EB cell by smRNA FISH. Left, raw image. Right, foci detection (green squares) with ImageJ Macro. **c** Average number of mRNA molecules/cell determined by smRNA FISH for *Scl, Mesp1* and *Tbx6*, from day 3 to 4.5 EBs. Cell no.: number of cells analysed at each time point. **d** Distribution of *Scl*-positive cells (*Scl*/*Mesp1*/*Tbx6, Scl*/*Tbx6*, *Scl*/*Mesp1*, *Scl-only*) in day 3, 3.5, 4, and 4.5 EBs. Total number of EB cells and percentage of *Scl*-positive cells are indicated for each timepoint. Cells are considered positive for a marker when harbouring 6 or more foci. **e** smRNA FISH images showing a negative cell (top panel) and a cell positive for *Scl, Mesp1 and Tbx6* (bottom panel) from day 3.5 EBs. Arrows indicate typical foci for each mRNA species; white star, background signal. **f** Significant non-linear negative correlation of expression between *Scl* and *Mesp1* and *Scl* and *Tbx6*. Each dot represents a cell from day 3.5 EBs (total cells: 734). *X*-axis, number of *Scl* foci per cell; *Y*-axis, *Mesp1* foci*;* *Z*-axis*Tbx6* foci. Numbers of *Tbx6* foci are also indicated by a grey-red scale. Examples of *Scl/Mesp1* and *Scl/Tbx6* negatively correlated cells (i, ii, iii; Fig. 1g) are marked. Correlation coefficients: *Scl/Mesp1*: −0.2379, *p*-value < 0.0001, 95% confidence interval (CI): −0.3117/−0.1612; *Scl/Tbx6*: −0.1504, *p*-value < 0.002, 95% CI −0.2274/−0.07151. N, L, H: number of *Scl* foci/cell (N, negative; L, low (6–20 foci); H, high (21–139 foci)). **g** smRNA FISH images of representative cells showing *Scl*^*high*^/*Mesp1*^low^ (i, left) and *Scl*^*high*^/*Tbx6*^low^ (i, right), *Scl*^*low*^*/Mesp1*^high^ (ii), *Scl*^*low*^*/Tbx6*^*high*^ (iii) mRNA foci. Scale bars: 11.3 μm. See also Supplementary Fig. 2

Mouse ES cell/embryoid body (EB) differentiation cultures recapitulate major embryonic developmental processes^19^ (Fig. 1a, top). Following production of *Fgf5*^+^ epiblast-like cells, *Brachyury+* mesoderm develops from day 2.5 (Fig. 1a, right). From day 3, expression of VEGFA receptor, *Flk1*, marks the emergence of mesodermal progenitors at the origin of the endothelial and blood lineages^20^. Day 3/3.5 sees robust expression of tissue-specific regulators of distinct mesoderm-derived lineages: *Scl* (haematopoietic^5^), *Mesp1* (cardiac^21^) and *Tbx6* (paraxial^22^) (Fig. 1a, bottom). This stage corresponds to the development of nascent/posterior mesoderm in the primitive streak of day E7/7.5 mouse embryos (Supplementary Fig. 1) and marks the onset of lineage specification in the ES/EB model.

To test if multilineage-primed mesodermal progenitors exist, we asked if *Scl, Mesp1 and Tbx6* were co-expressed in the same cells by single molecule mRNA (smRNA) FISH. We designed probe libraries for each mRNA species, co-stained day 3 to day 4.5 EB cells and quantitated the number of single mRNA molecules (foci) in individual cells (Fig. 1b). The average foci number/cell for each mRNA target (Fig. 1c) followed the expression pattern of the corresponding mRNA species in cell populations (Fig. 1a, bottom). When assessing co-expression of the three markers, we observed triple (*Scl/Mesp1/Tbx6*) and double (*Scl/Mesp1*, *Scl/Tbx6*) positive cells (Fig. 1d, e, Supplementary Fig. 2a–c). The proportion of these cells amongst *Scl*-expressing cells increased from day 3 to day 3.5 (20–35% of total *Scl-*positive cells, Fig. 1d), but decreased thereafter (9 and 3% at days 4 and 4.5, Fig. 1d). Because *Mesp1* broad expression in early gastrulating embryos suggests it could label mesodermal lineages other than cardiac^23,24^, data obtained with *Mesp1* were confirmed with another cardiac-defining marker, *Gata4* (Supplementary Fig. 2d). The high proportion of *Scl/Mesp1*/*Gata4*-coexpressing cells amongst day 3.5 *Scl/Mesp1-*positive cells (87%) validated *Mesp1* as a marker of the cardiac lineage in *Scl*-expressing cells, at the developmental timepoint examined. Thus, *Scl, Mesp1* and *Tbx6* are co-expressed over a tight developmental time-window. At a single cell level, the decrease in double and triple positive cells in day 4/4.5 EBs was accompanied by increased numbers of *Scl*-expressing cells (45 and 51% of total EB cells).

We then correlated the level of *Scl*, *Mesp1* and *Tbx6* expression in day 3.5 single cells. High (H) numbers of *Scl* foci (21–139) were preferentially associated with no (0–5) or low (6–20 foci) expression of *Mesp1* or *Tbx6* (example cell (i), Fig. 1f, g, Supplementary Fig. 2e), whilst highest numbers of *Mesp1* (21–166) or *Tbx6* (21–40) foci preferentially occurred in cells negative (N) for *Scl* (cells (ii) and (iii), Fig. 1f, g). Similarly, we never detected co-expression of the three genes at high levels (Supplementary Fig. 2f).

In conclusion, co-expression of *Scl/Mesp1/Tbx6*, *Scl/Mesp1* and *Scl/Tbx6* preferentially occurs at low mRNA levels in a subset of *Scl*-expressing cells at day 3/3.5, consistent with multi-lineage-priming of distinct mesodermal lineages. Subsequently, as *Scl* mRNA levels increase, reflecting consolidation of the blood programme, expression of alternative lineage-specific regulators is lost.

### Immunophenotypic conversion of *Scl*^*−/−*^ FLK1 positive cells

**Fig. 2:**
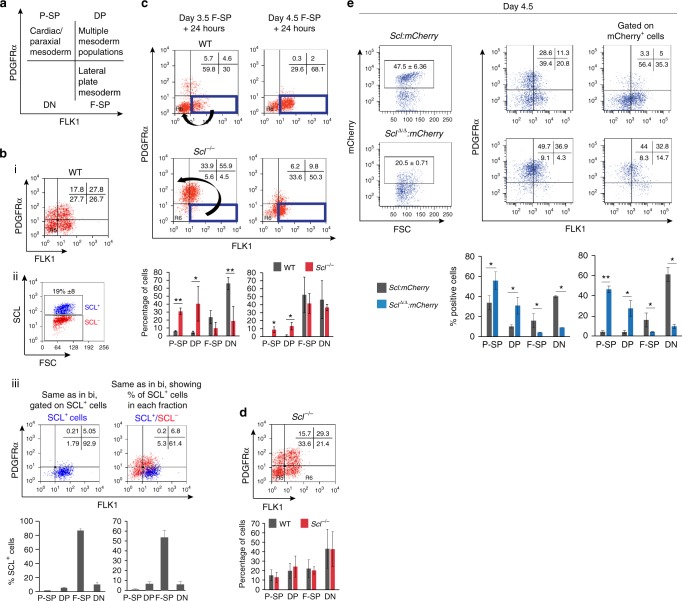
Immunophenotypic conversion of *Scl*-null FLK1-single positive cells. **a** Days 3.5/4 ES cell-derived mesodermal populations are functionally defined by expression of cell surface markers FLK1 and PDGFRα. P-SP, PDGFRα single positive; DP, double positive; F-SP, FLK1 single positive; DN, double negative. **b** (i) Distribution of FLK1- and PDGFRα−positive populations in WT day 3.5 EBs. (ii) SCL protein expression in day 3.5 WT EBs (intra-cellular FACS). (iii) Left, top, distribution of FLK1/PDGFRα-positive cells shown in bi and gated on SCL-positive cells; bottom, mean of 9 independent experiments; Right, top, distribution of SCL^+^ cells in each FLK1/PDGFRα compartment. Blue events: SCL^+^ cells, red events: SCL^-^ cells; bottom, mean of 9 independent experiments. **c** Re-aggregation assays. F-SP populations were FACS-sorted from WT and *Scl*^*-/-*^ EBs (left panels, day 3.5; right panels, day 4.5), allowed to re-aggregate for 24 h and analysed for FLK1/PDGFRα expression. The arrows show the different immunophenotypic conversions of WT and *Scl*^*-/-*^ cells at day 3.5 + 24 h. Bottom, mean of two independent experiments. **d** Top, distribution of FLK1-positive and PDGFRα-positive populations in *Scl*^*-/-*^ day 3.5 EBs; bottom, comparison with WT cells (shown in bi), mean of 4 independent experiments. **e** *Scl:mCherry* and *Scl*^*Δ/Δ*^:mCherry reporter lines analysed in day 4.5 EBs. Representative FACS plots of mCherry expression (left), FLK1/PDGFRα expression (middle) and FLK1/PDGFRα plots gated on mCherry-positive cells (right) are shown. Below, mean of 2 independent experiments. Mean ± SD is shown in **b**–**e**; student’s *t*-test, *p < 0.05, **p < 0.01. Scale bars, 100 μm. See also Supplementary Fig. 3

Having established the presence of multi-lineage primed cells, we next investigated how lineage potentials resolve. We studied blood fate selection in day 3.5 EBs, when 35% of *Scl*-expressing cells co-express *Mesp1* and/or *Tbx6* mRNA. To identify lineage output, we monitored expression of cell surface markers FLK1 and PDGFRα that separate mesodermal populations with distinct functional potential^25–28^ (Fig. 2a). Multiple mesodermal potentials are initially found in the FLK1^+^PDGFRα^+^ (double positive, DP) compartment. FLK1 single positive (F-SP) cells contain lateral plate mesoderm with haematopoietic/endothelial potential and PDGFRα single positive (P-SP) cells contain paraxial and cardiac potential.

Flow cytometry staining for FLK1, PDGFRα (Fig. 2bi, Supplementary Fig. 3a) and SCL (Fig. 2bii) showed that 19% (±8) of day 3.5 EB cells expressed SCL. 80–93% of SCL-expressing cells were in the F-SP lateral plate compartment (Fig. 2biii, left panels). Conversely, 50–62% of the F-SP population expressed SCL (Fig. 2biii, right panels). To study the fate of SCL-expressing cells, we FACS-sorted day 3.5 F-SP cells (Supplementary Fig. 3b), re-aggregated the cells into EBs and, after 24 h, re-analysed FLK1/PDGFRα expression (Fig. 2c, left, wild-type (WT) panel). 60–70% of the F-SP cells differentiated into FLK1^-^PDGFRα^-^ (DN, double negative) maturing haematopoietic cells^29,30^. However, about 10% of F-SP cells acquired PDGFRα expression and were detected in the DP/P-SP compartments. These PDGFRα^+^ cells may represent rare cells with higher expression of *Mesp1* and *Tbx6* than *Scl* (*Scl*^*low*^*Mesp*^*hi*^*, Scl*^*low*^*Tbx6*^*hi*^ and *Scl*^*low*^*Mesp*^*hi*^*Tbx6*^*hi*^ - Supplementary Fig. 2e–f), suggesting there may be competition between blood and non-blood fates within multilineage-primed cells, dependent on *Scl, Mesp1* and *Tbx6* levels. Therefore, absence of SCL might increase the number of cells adopting alternative fates.

To test this hypothesis, we isolated F-SP cells from *Scl*^*−/−*^ day 3.5 EBs (Fig. 2d) and subjected them to the same re-aggregation assay employed with WT cells. After 24 h, *Scl*^*−/−*^ F-SP cells exhibited a markedly different immunophenotypic pattern (Fig. 2c, left, *Scl*^*−/−*^ panels). The majority of *Scl*^*−/−*^ F-SP cells acquired PDGFRα expression and were DP or P-SP, consistent with the hypothesis that they may have acquired non-blood fates.

To validate these observations, we generated two *Scl:mCherry* reporter ES cell lines expressing either WT SCL (*Scl:mCherry*) or a non-functional form of SCL missing the vital basic helix-loop-helix domain^31^ (*Scl*^*Δ/Δ*^*:mCherry*, Supplementary Fig. 3c–f). Immunophenotyping of day 4.5 EB cells showed that 47.5% of *Scl:mCherry* cells and 20.5% of *Scl*^*Δ/Δ*^*:mCherry* were mCherry^+^ (Fig. 2e, left panels); this difference is likely due to SCL’s positive transcriptional auto-regulation^32^. Importantly, a dramatic 76.8% of *Scl*^*Δ/Δ*^*:mCherry*^*+*^ cells expressed PDGFRα (DP/P-SP compartments), as opposed to 8.3% of *Scl:mCherry*^*+*^ cells (Fig. 2e, right panels), confirming our earlier results.

A key observation from the smRNA FISH experiments was the tight temporal control of transcriptional priming of blood, cardiac and paraxial lineages, peaking at day 3.5 and greatly reduced by day 4.5 (Fig. 1d, Supplementary Fig. 2b). This suggested that cells could alter mesodermal fates maximally at day 3.5. Indeed, when compared to day 3.5 *Scl*^*−/−*^ F-SP cells, a greatly decreased number of day 4.5 *Scl*^*−/−*^ F-SP cells acquired low level PDGFRα expression in the re-aggregation assay (Fig. 2c, right panels). Therefore, temporal restriction of multi-lineage priming correlates with the tight temporal window in which F-SP cells acquire PDGFRα expression.

### Absence of SCL increases adoption of alternative fates

**Fig. 3:**
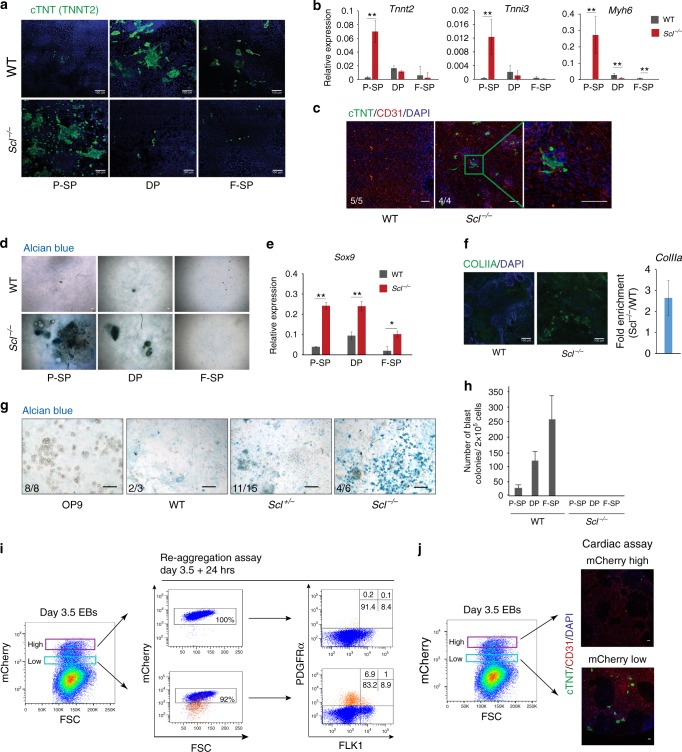
*Scl*-null cells acquire cardiac and paraxial potentials. **a**–**c** Cardiac assays. **a** Day 3.5 WT and *Scl*^*-/-*^ P-SP, DP, and F-SP populations were replated in cardiac condition for 7 days and cTNT (cardiac troponin) expression monitored by immunofluorescence (IF); scale bar, 100 μm. **b** RT-qPCR analysis of cardiac gene expression (*Tnnt2*, *Tnni3* and *Myh6*) relative to *Gapdh* in cultures shown in **a**; *n* = 3–5. **c** Day E9.5 WT and *Scl*^-/-^ mouse yolk sacs replated in cardiac assay for 7 days. IF reveals cardiomyocytes (cTNT, green), endothelium (CD31, red) and nuclei (DAPI, blue). **d**–**g** Chondrogenic assays. **d** Day 3.5 WT and *Scl*^*-/-*^ P-SP, DP and F-SP populations were replated in chondrogenic condition for 21 days. Alcian blue staining reveals glycosaminoglycan clusters; **e** RT-qPCR analysis of *Sox9* expression relative to *Gapdh* in cultures shown in **d**, *n* = 2. **f** Collagen IIa (ColIIa) expression following culture of day 3.5 WT and *Scl*^*-/-*^ EB cells in chondrogenic condition (left, IF: COLIIA green, DAPI blue; right, RT-qPCR analysis) *n* = 2. **g** Alcian blue staining of day 18 chondrogenic cultures from day E9.5 WT, *Scl*^*+/-*^ and *Scl*^*-/-*^ mouse yolk sacs. OP9, no yolk sac cells. **c**, **g** Number of embryos presenting the phenotype shown is indicated for each genotype. **h** Blast colony assay showing number of endothelial/haematopoietic colonies obtained from day 3.5 WT and *Scl*^*-/-*^ purified mesodermal populations (P-SP, DP, F-SP); *n* = 3. **i** Left: day 3.5 *Scl:mCherry* WT cells were FACS-sorted according to the level of mCherry (and therefore SCL) expression into low and high fractions. Right: mCherry^high^ and mCherry^low^ cells were re-aggregated for 24 h, and mCherry (left) and FLK1/PDGFRα (right) expression re-assessed. Note that only the day 3.5 mCherry^low^ fraction produced a PDFGRα^+^ population (bottom right panel, orange events). At day 3.5 + 24 h, the majority of the PDGFRα^+^ cells have lost mCherry expression (bottom left panel, orange events). **j** Day 3.5 *Scl:mCherry*^*high*^ and *Scl:mCherry*^*low*^ FACS-sorted cells were replated in cardiac assay. IF reveals cardiomyocytes (cTNT, green), endothelium (CD31, red) and nuclei (DAPI, blue). Mean ± SD is shown (**b**), (**e**), (**f**), (**h**); student’s *t*-test, *p < 0.05, **p < 0.01. Scale bars, 100 μm. See also Supplementary Fig. 3

We next asked if gain in PDGFRα expression in absence of SCL reflected increased numbers of cells adopting non-blood cell fates. We first replated day 3.5 WT and *Scl*^*−/−*^ mesodermal populations (P-SP/DP/F-SP) in conditions promoting cardiac development. Cardiac cell output was assessed by immunofluorescence staining for cardiac troponin (cTNT, Fig. 3a) and mRNA analysis (*Tnnt2*/*Tnni3/Myh6*, Fig. 3b). Strikingly, significantly more cardiac cells were detected in the *Scl*^*−/−*^ vs. WT P-SP populations, showing a rapid functional conversion of *Scl*^*−/−*^ cells. We confirmed ectopic cardiomyocyte production in vivo from *Scl*^*−/−*^ mouse embryos. Obvious foci of cardiac cTNT^+^ cells were detected in day E9.5 *Scl*^*−/−*^, but not WT, yolk sacs (Fig. 3c). Next, we tested the paraxial potential of WT and *Scl*^*−/−*^ cells by re-plating day 3.5 fractionated or whole EB cells in chondrogenic conditions. Cultures from *Scl*^*−/−*^ P-SP/DP populations exhibited increased formation of chondrogenic-specific glycosaminoglycan clusters (Alcian blue staining, Fig. 3d), and increased expression of the early chondrogenic marker, *Sox9*^33^, (Fig. 3e), when compared to WT cells. Similarly, cultures from *Scl*^*-/-*^ EB cells showed more cells positive for COLLAGEN IIA, major downstream target of *Sox9*^34^, and increased *ColIIa* mRNA expression (Fig. 3f). Increased chondrogenic potential was confirmed in vivo from *Scl*-null yolk sacs (Fig. 3g). As controls, haematopoietic cells developed only from WT, but not *Scl*^*−/−*^, F-SP/DP populations, in blast colony assays^35^ (Fig. 3h). Therefore, in absence of SCL, functional cardiac and paraxial PDGFRα^+^ populations expand in day 3.5 EBs.

Finally, to further validate the hypothesis that only *Scl*^low^ expressing cells can adopt a non-blood fate, we purified day 3.5 *Scl:mCherry*^*low*^ and *Scl:mCherry*^*high*^ cells and tested their developmental potential in re-aggregation and cardiac assays. As expected, 24 h after purification, the majority of the *mCherry*^*low*^ cells became *mCherry*^*high*^ and FLK1/PDGFRα DN, pursuing their normal hematopoietic differentiation trajectory (Fig. 3i, Supplementary Fig. 3g). Remarkably, however, day 3.5 *Scl:*mCherry^*low*^, but not *Scl:mCherry*^*high*^, cells generated a PDGFRα-positive population in the re-aggregation assay (7.9%, Fig. 3i). Confirming the adoption of alternative fates by cells expressing low levels of *Scl*, cTNT-positive cardiomyocytes were exclusively observed from *Scl:mCherry*^*low*^ cells in cardiac cultures (Fig. 3j). Therefore, cell fate decisions critically depend on *Scl* mRNA thresholds.

In conclusion, our data identify a temporally restricted conversion of day 3.5 *Scl*^*−/−*^ F-SP cells into functional cardiac cells, in line with previous data showing ectopic emergence of PDGFRα cells with a cardiac expression programme in *Scl*^*−/−*^ yolk sac^10^. We extend these observations by showing that paraxial lineage cells are also produced from day 3.5 *Scl*^*−/−*^ F-SP cells. Critically, the tight time-window during which non-blood lineages are produced in absence of SCL coincides with the restricted developmental period revealing multilineage-primed mesodermal cells.

### SCL controls expression of transcriptional repressors

**Fig. 4:**
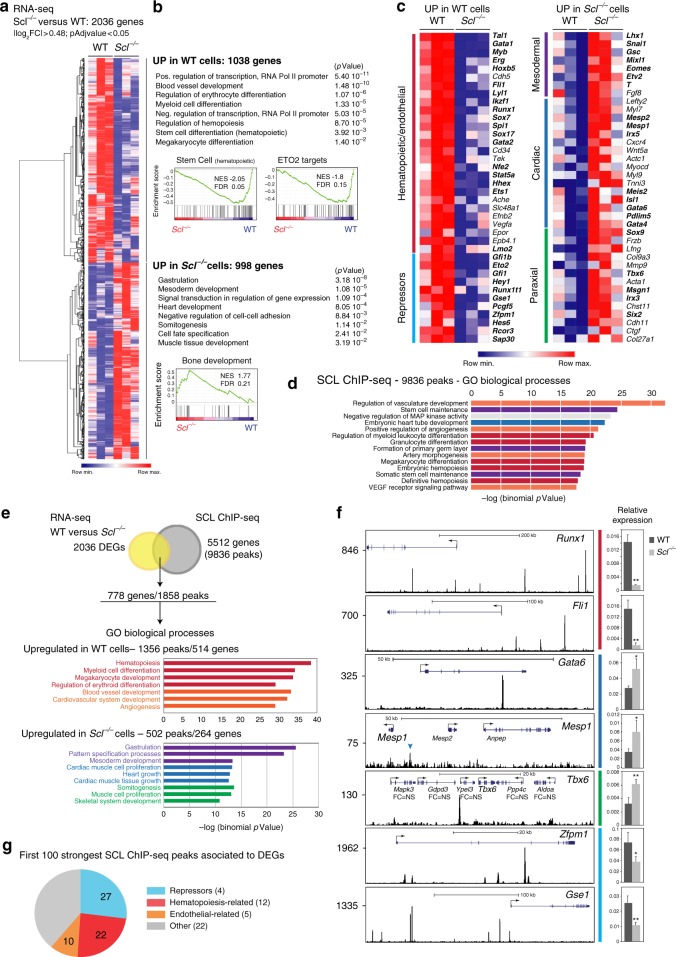
SCL controls distinct gene regulatory networks in day 3.5 FLK1^+^ cells. **a** Hierarchical clustering of RNA-seq data from day 3.5 WT and *Scl*^*-/-*^ FLK1^+^ EB cells. **b** Gene ontology (GO) processes associated to differentially expressed genes (DEGs) (PANTHER and GSEA analyses). **c** Heatmap showing selected DEGs associated to GO processes identified in **b**. In bold, transcriptional regulators. **d** The top more significant GO biological processes associated to SCL-bound loci (GREAT analysis). **e** Integration of SCL ChIP-seq and RNA-seq data reveals 778 SCL direct differentially expressed target genes. Below, GO terms attributed to DEG-associated peaks (GREAT analysis). **f** Left, SCL ChIP-seq tracks of selected direct DEGs; FC = NS, fold-change in expression in *Scl*^*-/-*^ cells is not significant (RNA-seq data). *Tbx6* locus: the SCL peak was attributed to *Tbx6*, the closet DEG. Right, RT-qPCR gene expression analysis relative to *Gapdh* from WT and *Scl*^*-/-*^ day 3.5 FLK1^+^ cells. *n* = 3, mean ± SD; student’s *t*-test, *p < 0.05, **p < 0.01. Colour code, same as in **c**. **g** Biological functions attributed to the 100 strongest DEG-associated SCL ChIP-seq peaks. In brackets, numbers of genes in each category. See also Supplementary Data 1, 2

We next defined the molecular mechanism leading to acquisition of non-blood cell fates in absence of SCL. RNA sequencing revealed 2036 differentially expressed genes (DEGs) between day 3.5 WT and *Scl*^*−/−*^ FLK1^+^ cells (Fig. 4a). Consistent with the functional cellular output (Fig. 3a–h), the 1038 genes normally activated by SCL (upregulated in WT versus *Scl*^*−/−*^ cells) were associated with haematopoietic/endothelial differentiation, as revealed by Gene Ontology (GO) terms and GSEA analyses (Fig. 4b, examples of genes in Fig. 4c). In contrast, the 998 genes upregulated in *Scl*^*-/-*^ cells were associated with mesoderm, cardiac and paraxial lineage development (Fig. 4b, examples of genes in Fig. 4c). Interestingly, genes encoding transcriptional repressors were also activated in WT cells (Fig. 4b, c), for example SCL’s partner ETO2 (CBFA2T3)^36^.

SCL ChIP-seq from day 3.5 WT FLK1^+^ cells revealed 9836 SCL-bound loci. GO terms associated with the genes nearest to the peaks were related to haematopoiesis, vasculature and cardiac development (Fig. 4d). Combining RNA- and ChIP-seq datasets revealed 778 differentially expressed, SCL-bound loci (hereafter referred to as SCL direct DEGs, Fig. 4e, Supplementary Data 1). 514/778 genes were more highly expressed in WT cells and related to haematopoiesis and vasculogenesis (*Runx1*/*Fli1*, Fig. 4e, f.) 264/778 genes showed increased expression in *Scl*^*−/−*^ cells and were related to mesoderm, cardiac (*Gata6*/*Mesp1)* and paraxial lineages (*Tbx6*) (Fig. 4e, f).

We next examined the 100 DEG-associated peaks showing the strongest SCL binding (greatest number of mapped reads from ChIP-seq data) (Fig. 4g, Supplementary Data 2). Remarkably, 97 of these peaks were attributed to genes normally activated by SCL. Whilst 22 peaks were associated to haematopoiesis-related genes and 10 to endothelium-related genes, 27 were associated with 4 genes encoding blood-related transcriptional repressors: *Zfpm1* and *Gse1* (Fig. 4f), *Eto2* and *Runx1t1 (Eto)*. In addition to these 4 genes, another 7 transcriptional repressors, either expressed in blood cells (*Zfpm2*/*Gfi1*/*Gfi1b*/*Ikzf1*/*Izkf2)* or members of the general repressive complex ncPRC1 (*Rybp* and its partner *Pcgf5*) were SCL direct targets (Supplementary Data 1). Of note, the number of SCL peaks was higher in loci normally activated (*Runx1/Fli1/Zfpm1/Gse1*) than on those normally repressed (*Gata6*/*Mesp1*/*Tbx6*) (Fig. 4f; Wilcoxon rank-sum test confirmed the difference in peak distribution between all 514 downregulated and 264 upregulated direct DEGs at *p* value 5.8 × 10^−08^). This suggests mechanistic differences in SCL-mediated transcriptional activation versus repression.

In summary, SCL normally activates a blood/endothelial programme in FLK1^+^ cells and represses cardiac and paraxial programmes. Unexpectedly, SCL strongly binds to and activates expression of genes encoding transcriptional repressors. This suggests that SCL may suppress cardiac/paraxial programmes in blood-fated cells through transcriptional activation of repressors.

### SCL controls levels of histone marks linked to repression

**Fig. 5:**
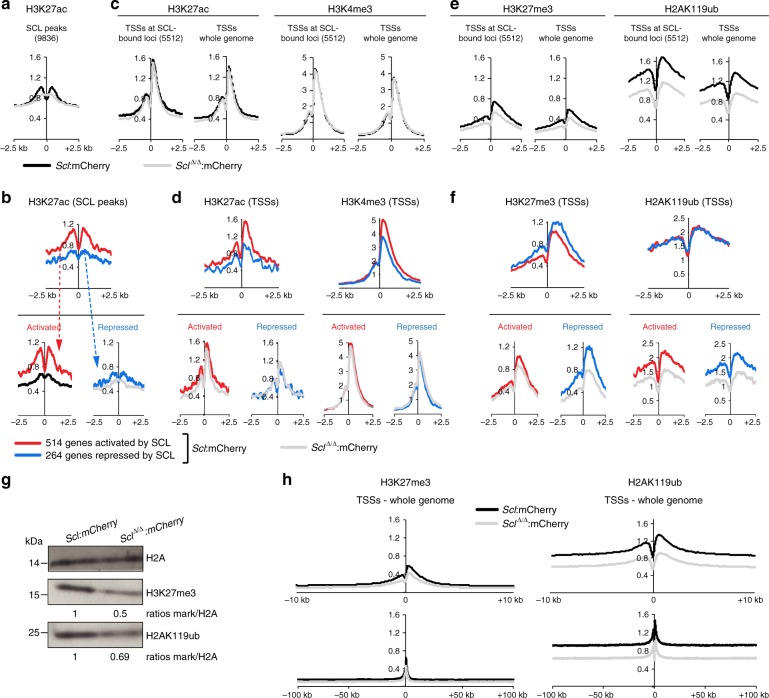
SCL loss-of-function affects the epigenetic landscape of blood-fated progenitors. **a**, **c**, **e** Distribution plots of normalised histone mark ChIP-seq signals in mCherry^+^ cells purified from *Scl:mCherry* and *Scl*^*Δ/Δ*^*:mCherry* day 4 EBs. Signals are sorted on SCL ChIP-seq peaks (**a** H3K27ac only), TSSs of SCL-bound 5512 genes and TSSs across the whole genome, ±2.5 kb, (**c** activation associated-marks H3K27ac and H3K4me3; **e** repression associated-marks H3K27me3 and H2AK119ub). **b**, **d**, **f** Distribution plots of normalised histone mark ChIP-seq signals in mCherry^+^ cells purified from *Scl:mCherry* and *Scl*^*Δ/Δ*^*:mCherry* day 4 EBs on SCL direct DEGs (514 activated genes (red) and 264 repressed genes (blue)). Signals are sorted on SCL ChIP-seq peaks associated to SCL direct DEGs (**b** H3K27ac only) and TSSs of SCL direct DEGs, ± 2.5 kb (**d**, activation associated-marks; **f** repression associated-marks). Top, *Scl:mCherry* EB cells; bottom, *Scl:mCherry* and *Scl*^*Δ/Δ*^*:mCherry* EB cells. **g** Western blot analysis of H3K27me3 and H2AK119ub levels in mCherry^+^ cells purified from *Scl:mCherry* and *Scl*^*Δ/Δ*^*:mCherry* day 4 EBs. H2A, loading control. Relative levels of each histone mark over H2A in mutant versus control cells are indicated. *n* = 2. **h** Distribution plots of normalised H3K27me3 and H2AK119ub ChIP-seq signals in mCherry^+^ cells purified from *Scl:mCherry* and *Scl*^*Δ/Δ*^*:mCherry* day 4 EBs. Signals are sorted on TSSs across the whole genome; top, ±10 kb, bottom, ±100 kb. See also Supplementary Figs. 4, 8

To further understand SCL-driven mechanisms of transcriptional regulation, we surveyed histone marks associated with activated and repressed genes (H3K27ac/H3K4me3 and H3K27me3/H2AK119ub, respectively) by quantitative ChIP-seq (ChIP-Rx^37^) in mCherry^*+*^ cells isolated from *Scl:mCherry* and *Scl*^*Δ/Δ*^*:mCherry* day 4 EB cells. H3K27ac, the only mark detected at SCL genomic binding sites, was analysed at all 9836 SCL ChIP peaks (Fig. 5a) and on SCL peaks associated to SCL’s 778 direct DEGs (Fig. 5b). As all these histone modifications mark promoter regions, we examined their distribution at transcriptional start sites (TSSs) of SCL-bound 5512 loci, whole genome TSSs and TSSs of SCL’s 778 direct DEGs (Fig. 5c–f).

H3K27ac was higher at SCL 9836 peaks in WT cells (*Scl:mCherry*) than in *Scl-null* cells (*Scl*^*Δ/Δ*^*:mCherry*) (Fig. 5a), consistent with SCL’s ability to recruit histone acetyltransferase P300^38^. H3K27ac at SCL peaks located in SCL direct DEG genomic loci was higher in the 514 activated genes compared to the 264 repressed genes (Fig. 5b, top) in an SCL-dependent manner (Fig. 5b, bottom). In contrast, H3K27ac and H3K4me3 levels at TSSs of the 5512 loci bound by SCL (of which only 422 are bound by SCL at TSSs) and TSSs genome-wide were SCL-independent (Fig. 5c). As expected, the level of these two histone marks was higher at TSSs of the 514 activated genes compared to the 264 repressed genes and SCL-independent (Fig. 5d). Extending these observations, we noticed a broad and punctuate H3K27ac pattern at activated gene loci, not just restricted to SCL peaks and fully SCL-dependent (*Fli1*/*Mafb*, Supplementary Fig. 4a). SCL-independent H3K4me3 distribution at TSSs was visualised at both activated (*Fli1*/*Mafb*) and repressed (*Gata6*/*Irx3)* genes (Supplementary Fig. 4a, b). In summary, levels of H3K27 acetylation are higher and SCL-dependent at SCL’s peaks associated to genes normally activated. At TSSs, both H3K27ac and H3K4me3 marks are more prominent at genes normally activated and are SCL-independent.

We next studied histone marks associated with repression, H3K27me3 and H2AK119ub. In contrast to H3K27ac and H3K4me3, we observed a striking, global SCL-dependent reduction of these marks, at TSSs of the 5512 SCL-bound loci and TSSs genome-wide (Fig. 5e). This reduction was confirmed by western blot (Fig. 5g, Supplementary Fig. 8a). Furthermore, whereas SCL-dependent levels of H3K27me3 were just confined to ~2.5 kb around TSSs genome-wide, SCL-dependent levels of H2AK119ub extended for large domains (at least 100 kb) either side of the TSSs (Fig. 5h). Focussing on the 778 SCL direct DEGs, H3K27me3 levels were higher at TSSs of repressed, rather than activated, genes (Fig. 5f, top), and showed SCL-dependence at repressed and, to a lesser extent, activated genes (Fig. 5f, bottom; *Gata6*/*Irx3*/*Mafb*, Supplementary Fig. 4). Surprisingly, the level of the H2AK119ub mark did not vary between TSSs of genes repressed and activated by SCL (Fig. 5f, top), and, in both cases, was SCL-dependent (Fig. 5f, bottom, Supplementary Fig. 4).

Taken together, these data show that SCL mediates H3K27 acetylation specifically at SCL-bound *cis*-regulatory elements of genes it normally activates. SCL also regulates the level of repression-associated histone marks, H3K27me3 and H2AK119ub, not only at TSSs of genes it normally represses but also at some (H3K27me3) or all (H2AK119ub) genes it activates. This suggests that inappropriate gene activation may be restrained by SCL-mediated repressive environment. Regulation of repression, as surveyed by histone modifications, constitutes an important facet of SCL function at the onset of blood specification.

### ETO2, RYBP and SCL are functionally linked

To investigate SCL-regulated active repression of gene expression in mesoderm patterning, we studied two transcriptional repressors and direct SCL target genes in day 3.5 FLK1^+^ cells, ETO2 and RYBP. ETO2 is a known SCL partner that represses premature megakaryocytic and erythroid gene activation later in development^36,39,40^. RYBP, member of non-canonical PRC1 (ncPRC1) complexes, modulates H2AK119 ubiquitination levels through stabilisation of RING1B recruitment and enhancement of its enzymatic activity^41–43^. Given the role of PcG complexes in development and the changes in ubiquitination levels observed in *Scl*^*-/-*^ cells, we reasoned that RYBP may mediate some of SCL-regulated repression mechanisms.

**Fig. 6:**
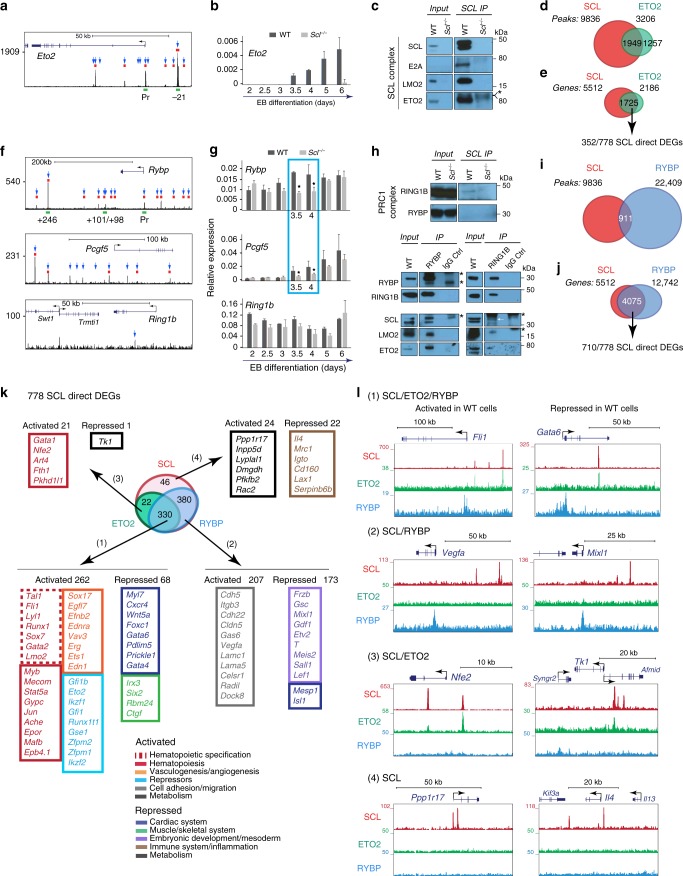
Genome-wide binding of SCL, ETO2 and RYBP in FLK1^+^ cells. **a** SCL ChIP-seq track of *Eto2* locus in day 3.5 FLK1^+^ EB cells. Blue arrows, SCL peaks associated to *Eto2*; red rectangles, ATAC peaks; green rectangles, SCL-bound *cis*-elements further detailed in Supplementary Fig. 5. **b** RT-qPCR analyses of *Eto2* mRNA expression from WT and *Scl*^*-/-*^ EB differentiation kinetics (day 2–day 6) relative to *Gapdh*. *n* = 3–5, mean ± SD; student’s *t*-test *p < 0.05. **c** Western blot analysis of day 4.5 WT EB nuclear extracts immunoprecipitated (IP) with anti-SCL antibodies. Members of SCL complex are detected as indicated. *n* = 3. **d** Overlap between SCL and ETO2 ChIP-seq peaks. **e** Overlap between SCL-bound and ETO2-bound genes. 352 of these are SCL direct DEGs. **f** SCL ChIP-seq track of *Rybp, Pcgf5 and Ring1b* loci in day 3.5 FLK1^+^ EB cells. Blue arrows, SCL peaks associated to the genes; red rectangles, ATAC peaks; green rectangles, SCL-bound *cis*-elements further detailed in Supplementary Fig. 5. **g** RT-qPCR analyses of *Rybp, Pcgf5 and Ring1b* mRNA expression from WT and *Scl*^*-/-*^ EB differentiation kinetics (day 2–day 6) relative to *Gapdh*. *n* = 3–5, mean ± SD; student’s *t*-test *p < 0.05. **h** Western blot analysis of day 4.5 WT EB nuclear extracts immunoprecipitated (IP) with anti-SCL antibodies (top), -RYBP (bottom, left) and -RING1B (bottom, right) antibodies. Members of PRC1 complex (RING1B, RYBP) and SCL complex (LMO2, ETO2) are detected as indicated. Asterisk (*) indicates heavy or light IgG chain. White arrow indicates SCL band in RING1B IP. **i** Overlap between SCL and RYBP ChIP-seq peaks. **j** Overlap between SCL-bound and RYBP-bound genes. Seven hundred and ten of these are SCL direct DEGs. **k** Overlap between SCL-bound, ETO2-bound, and RYBP-bound 778 SCL direct DEGs. Number and representative examples of SCL direct target genes in the four categories defined by SCL, ETO2, and RYBP binding are shown. **l** UCSC tracks showing SCL, ETO2 and RYBP binding on examples of activated and repressed SCL direct DEGs in the four categories defined by SCL, ETO2, and RYBP binding. See also Supplementary Figs. 4–6 and 9, Supplementary Data 3

The *Eto2* locus was bound by SCL at conserved *cis*-elements associated with open chromatin (ATAC-seq) and containing DNA-binding motifs associated to SCL binding (Fig. 6a, Supplementary Fig. 5a). *Eto2* mRNA expression was initiated at day 3.5, increased throughout EB differentiation, thus mirroring *Scl*’s expression (Figs. 1a, 6b) and was abolished in absence of SCL (Fig. 6b). Finally, ETO2 was immunoprecipitated from EB cells with SCL and partners E2A and LMO2 (Fig. 6c, Supplementary Fig. 9a).

Day 4 ETO2 ChIP-seq analysis showed co-localisation with SCL at 1949 peaks, which represent ~20% of all SCL peaks and 60% of all ETO2 peaks (Fig. 6d, Supplementary Fig. 6a). The biological processes related to the genes associated to these 1949 peaks were enriched for blood, vascular, cardiac and muscle development (Supplementary Fig. 6b). We then considered the 1725 gene loci co-occupied by SCL and ETO2. These corresponded to 31% of SCL-bound genes (1725/5512) and 79% of ETO2-bound genes (1725/2186) (Fig. 6e). These genes contained 352 out of SCL’s 778 direct DEGs (45%). The strength of SCL and ETO2 binding across these 352 DEGs was the same for both proteins: higher in the genes activated by SCL vs. those repressed (Supplementary Fig. 6c). Therefore, SCL and ETO2 often co-localise on activated and repressed genes and most likely co-operate in multi-protein complexes.

Similarly, SCL binding was detected at the loci encoding *Rybp* and its partner *Pcgf5*, in areas associated with open chromatin and SCL-binding DNA motifs (Fig. 6f, Supplementary Fig. 5b). Strikingly, expression of *Rybp* and *Pcgf5* was significantly downregulated in days 3.5/4 *Scl*^*−/−*^ EB cells (Fig. 6g), corresponding to the narrow time-window of transcriptional co-priming of blood/cardiac/paraxial lineages and developmental plasticity of F-SP cells (Figs. 1d, 2c). None of the other members of PRC1/PRC2 complexes we examined, including enzymes responsible for deposition of PcG-associated histone marks (*Ring1a/1b*, *Ezh1/2*), or members of the KAT and TrxG activating complexes, were direct targets of SCL (Fig. 6f, g, Supplementary Fig. 7), highlighting the unique relationship between SCL and RYBP/PCGF5. Finally, in contrast to the *Eto2* locus, binding of SCL to *Rybp* and *Pcgf5* loci was not seen in maturing blood cells (foetal liver pro-erythroblasts)^44^ (Supplementary Fig. 5a, b), suggesting specific requirements for these two repressors during early blood specification processes.

Immunoprecipitation (IP) of EB nuclear extracts with either RYBP or RING1B antibodies co-purified the SCL/E2A/LMO2 complex (Fig. 6h, bottom, Supplementary Fig. 9b, c). Reciprocal IP using SCL antibodies recovered RING1B but not RYBP (Fig. 6h, top, Supplementary Fig. 9a), suggesting that only a small fraction of RING1B/RYBP is complexed with SCL. Indeed, SCL and RYBP binding co-localised at only 911 peaks by day 3.5/4 ChIP-seq analyses, which represents 9% of SCL peaks and 4% of RYBP peaks (Fig. 6i, Supplementary Fig. 6d). Interestingly, GO analysis of the biological processes associated with the loci related to the 911 peaks showed enrichment for blood, cardiac and muscle development (Supplementary Fig. 6e). Finally, 4075 gene loci showed SCL and RYBP co-occupancy (where binding does not necessarily co-localise), corresponding to 73% of SCL-bound genes and 31% of RYBP-bound genes (Fig. 6j). These genes contained 710 out of 778 (91%) SCL direct DEGs, suggesting co-regulation by SCL and RYBP (Fig. 6j).

In conclusion, ETO2’s expression entirely relies on SCL throughout EB differentiation and full expression of RYBP and PCGF5 requires SCL within a narrow developmental window. ETO2 and RYBP interact with SCL. When considering gene loci, ETO2 co-occupies nearly half and RYBP the majority of SCL direct DEGs.

### Genome-wide interaction of SCL, ETO2 and RYBP with chromatin

To explore the shared and distinct gene sets regulated by SCL, ETO2 and RYBP, we examined the binding of these three regulators at SCL’s 778 direct DEGs. This defined 4 gene categories:

1. 330/778 gene loci (42%) were co-occupied by SCL/ETO2/RYBP (Fig. 6k, Supplementary Data 3). 262/330 genes were activated by SCL and required for blood (*Scl/Fli1/Runx1/Gata2)* and endothelial *(Sox17/Efnb2/Erg)* development, haematopoiesis and erythropoiesis (*Mafb/Gypc/Ache/Epor)*, and encoded transcriptional repressors (*Gfi1b/Eto2/Ikzf1/Runx1t1)* (Fig. 6k, l). Sixty-eight genes were repressed by SCL and involved in differentiation of cardiac (*Gata4/Gata6/Wnt5a/Foxc1)* and paraxial (*Irx3/Six2/Ctgf)* fates (Fig. 6k, l).
2. 380 genes (49%) co-occupied by SCL/RYBP, but not ETO2, were equally activated and repressed (Fig. 6k, l). Activated genes were mainly associated with cell adhesion/migration (*Cdh5/Itgb3/Cdh22/Lamc1*) and repressed genes with early developmental/mesoderm specification (*Gsc/Mixl1/Etv2/T)* (Fig. 6k, l).
3. 22 genes (3%) co-bound by SCL/ETO2, but not RYBP, were mainly activated but functionally required at later stages of haematopoietic differentiation (*Gata1/Nfe2)* (Fig. 6k, l).
4. 46 genes (6%) were only bound by SCL, equally activated or repressed and mainly involved in metabolism (activated genes) and inflammation (repressed genes) (Fig. 6k, l).

In conclusion, SCL and these repressors bind to overlapping and distinct functional categories of activated and repressed target genes.

### Loss of *Eto2* and *Rybp* phenocopies SCL-null cardiac phenotype

**Fig. 7:**
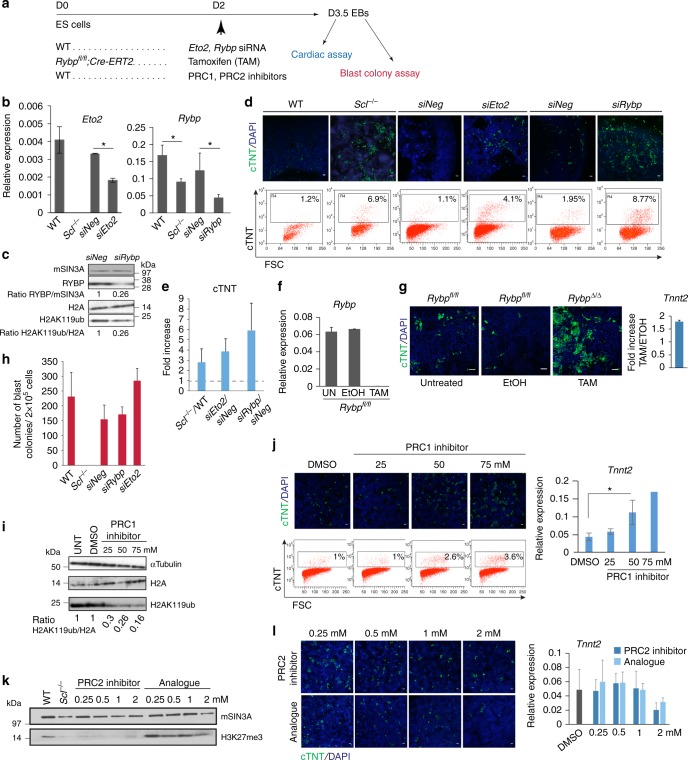
*Rybp* and *Eto2* knock-down phenocopies *Scl*^*-/-*^ cardiac phenotype. **a** Outline of functional assays. **b**–**e** siRNA-mediated *Eto2 (siEto2)* and *Rybp* (*siRybp*) knock-down in day 3.5 EBs. **b** qRT-PCR analysis of *Eto2* and *Rybp* mRNA levels. Analysis from day 3.5 WT and *Scl*^*-/-*^ EB cells is shown for comparison. *siNeg*, siRNA negative control; *n* = 4. **c** Western blot analysis of RYBP and H2AK119ub levels. mSIN3A and H2A, loading controls; *n* = 2. **d**, **e** Day 3.5 WT, *Scl*^*-/-*^, si*Eto2*-treated, si*Rybp*-treated and *siNeg*-treated EB cells were plated in cardiac condition. cTNT expression was monitored at day 7 by IF (**d**, top) and intra-cellular FACS (**d**, bottom). Quantitation of FACS data from *Scl*^*-/-*^, *siEto2*- and *siRybp*-treated cultures is shown as *Scl*^*-/-*^/WT, *siEto2*/siNeg and *siRybp*/siNeg ratios (**e**); *n* = 2. Scale bars, 100 μm. **f** qRT-PCR analysis of *Rybp* expression in day 3.5 untreated (UN), tamoxifen- (TAM) and ethanol (EtOH)-treated *CreERT2:Rybp*^*fl/fl*^ EBs; *n* = 2. **g** Day 3.5 untreated, EtOH-treated or TAM-treated *CreERT2:Rybp*^*fl/fl*^ cells were plated in cardiac condition. cTNT expression was assessed at day 7 by IF (green, left) and qRT-PCR analysis (*Tnnt2*, shown as TAM/ETOH fold increase, right). Scale bar, 100 μm. *n* = 2. **h** Day 3.5 WT, *Scl*^*-/-*^, si*Eto2*-treated, si*Rybp*-treated and *siNeg*-treated EB cells were plated in blast colony assay; *n* = 2. **i** Western blot analysis of H2AK119ub in day 3.5 EB cells treated with increasing concentrations of PRC1 inhibitor (PRT4165). UNT, untreated; αTubulin and H2A, loading controls, *n* = 2. **j** PRC1 inhibitor or DMSO-treated EBs were plated in cardiac assay, and cTNT expression assessed at day 7 by IF (green, top), FACS (bottom) and qRT-PCR (*Tnnt2*, right); *n* = 3. **k** Western blot analysis of H3K27me3 in day 3.5 EB cells treated with increasing concentrations of PRC2 inhibitor or analogue. mSIN3A, loading control, *n* = 2. **l** Day 3.5 EB cells treated with PRC2 inhibitor or analogue were plated in cardiac condition, and cTNT expression assessed at day 7 by IF (green, left) and qRT-PCR (*Tnnt2*, right). *n* = 2. Scale bars, 100 μm. Mean ± SD is shown in **b**, **f**, **h**, **j**, **l**; mean of ratios of mutant samples versus controls ± SD is shown in **e**, **g**; student’s *t*-test, *p < 0.05. See also Supplementary Fig. 8

Given the aggregate molecular and biochemical data that SCL, ETO2 and RYBP could function in concert at the time of blood specification, we asked if loss of ETO2 or RYBP could phenocopy loss of SCL in developing EBs (Fig. 7a). We first knocked down expression of *Eto2* and *Rybp* RNA and protein by siRNA, by 40–70%, at day 3.5 (*siEto2* and *siRybp*, Fig. 7b, c, Supplementary Fig. 8b). The level of reduction of *Rybp* mimicked that seen in absence of SCL during the window of mesodermal lineage specification (Fig. 6g) and, strikingly, led to decreased levels of H2AK119ub similar to that observed in *Scl*^*Δ/Δ*^*:mCherry* cells (Figs. 5g, 7c Supplementary Fig. 8c). Importantly, *Eto2* and *Rybp* knock-down cells produced a 4–6-fold increase in cTNT-positive cells in cardiac assays, similar to that observed in absence of SCL (Fig. 7d, e). Similarly, induction of *Rybp* deletion in *Rybp*^*fl/fl*^*:Cre-ERT2*^45^ EBs resulted in complete loss of *Rybp* expression (Fig. 7f) and expansion of cTNT-positive cardiac cells (Fig. 7g). Importantly, reduction of *Eto2* and *Rybp* expression did not alter blood lineage specification assayed by blast colony assays (Fig. 7h).

To directly test the function of H2AK119ub in cardiac development, we treated EBs with PRC1 ubiquitin ligase inhibitor PRT4165^46^. This achieved up to 84% decrease in ubiquitination levels (Fig. 7i, Supplementary Fig. 8d) and led to increased cardiac output, as judged by cTNT staining and *Tnnt2* mRNA expression (Fig. 7j), suggesting that H2AK119 ubiquitination mediates the cardiac phenotype observed from *Rybp* knock-down and *Scl-*null cells.

Finally, we asked if reduction in H3K27me3 could have a similar effect. We treated EBs with PRC2 inhibitor UNC1999 targeting EZH1/2 function^47^, to achieve near complete loss of H3K27me3 at day 3.5 (Fig. 7k, Supplementary Fig. 8e). Here, there was no increase in cardiac output assayed by cTNT staining and *Tnnt2* mRNA expression (Fig. 7l).

Altogether, these data suggest specific roles for ETO2 and RYBP and, by association, the PRC1 complex, most likely through H2AK119 ubiquitination, to suppress alternative fates in FLK1^+^ cells in collaboration with SCL.

## Discussion

**Fig. 8:**
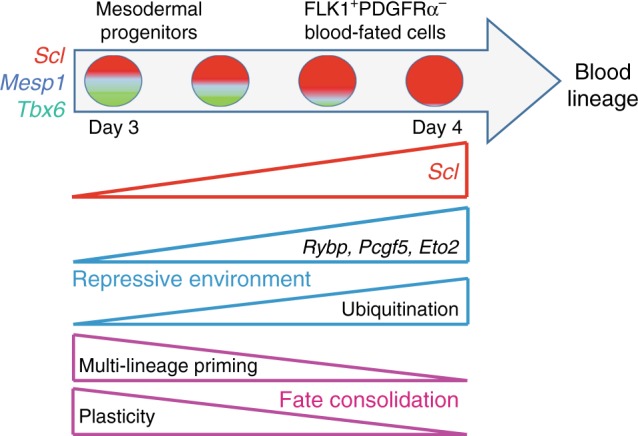
Model of SCL’s functions in blood lineage specification. Single mesodermal cells co-express blood (*Scl*), cardiac (*Mesp1*) and paraxial (*Tbx6*)-affiliated transcriptional regulators at low levels, establishing multi-lineage priming and cellular plasticity. As differentiation progresses, *Scl* levels increase in FLK1^+^PDGFRα^–^ cells. SCL establishes a global repressive environment by activating potent transcriptional repressors (*Rybp*/*Pcgf5*/E*to2)* and ensuring genome-wide, high H2AK119Ub levels. This leads to decreased *Mesp1* and *Tbx6* levels and repression of alternative fates. Activation of blood/endothelial gene expression programmes together with decreased plasticity allows consolidation of the blood fate and specification of the hematopoietic lineage

We have studied how lineage commitment is executed during development by focusing on how ES cell-derived FLK1^+^ mesodermal cells segregate into the blood lineage and suppress alternative cardiac and paraxial lineage programmes. We show that, at a narrowly defined time-point, a single TF, SCL, not only promotes haemopoiesis in FLK1^+^ cells, but concurrently suppresses mis-specification of these cells to alternative lineages (Fig. 8). A series of genome-wide molecular and biochemical studies demonstrates that SCL represses alternative gene expression in collaboration with ETO2 and the PRC1 complex at the precise time-point when multi-lineage transcriptional priming and cellular plasticity are detected. Whether this occurs in FLK1^+^SCL^+^ haematopoietic progenitors giving rise to primitive and/or definitive blood cells is not known as, to date, these two types of progenitors cannot be distinguished immunophenotypically or by gene expression.

Our findings have broad implications for our understanding of lineage determination. First, low level co-expression of *Scl, Mesp1* and *Tbx6* (required to specify blood, cardiac and paraxial lineages, respectively) confirms multi-lineage priming and suggests that progenitors can potentially adopt multiple fates. Existence of multilineage-primed cells agrees with studies showing that epiblast cells exhibit multipotency^48^ and with observations of “transcriptional noise” in day E6.5 mouse epiblast cells^49^. Importantly, multi-lineage priming needs to be distinguished from multi-lineage potency. Even though *Mesp1* and *Tbx6* are co-expressed with *Scl* in blood-fated FLK1^+^ cells, and, in absence of SCL, these cells can make cardiac and paraxial lineages, it is unclear if these cells are multi-potent at a clonal level in steady state, as multi-potency may only manifest in perturbed circumstances. One approach to address these open questions is to conduct in vivo fate tracing studies at appropriate developmental stages. This will characterise the extent of multi-potency and establish developmental relationships between mesodermal lineages.

Second, our data reveal the tight developmental window determining functional plasticity. Multi-lineage primed cells were most abundant in day 3.5 EBs and dramatically reduced at day 4.5. FLK1^+^ *Scl*^*−/−*^ cells gave rise to PDGFRα^+^ cardiac/paraxial cells at day 3.5, but not at day 4.5. Finally, SCL activated *Rybp* and *Pcgf5* expression at days 3.5/4 only. In wild-type cells, plasticity is detected in cells expressing low levels of *Scl*, as only *Scl:mCherry*^*low*^ cells can adopt a cardiac fate at day 3.5, likely to be elicited by higher *Mesp1* levels. This supports the notion of threshold-dependent fate determination, as recently reported for the TF ETV2 in hemangiogenic fate specification^50^. At earlier stages of development, the trophectoderm and the inner cell mass also exhibit time-restricted plasticity^51,52^. This is believed to establish flexible gene regulatory networks based on heterogeneity of gene expression^53^. Plasticity may therefore be a general property of cells undergoing lineage determination.

Third, our study reveals the importance of active repression of alternate fates in lineage-fated cells. To the best of our knowledge, we provide the first example of a single lineage-specific TF (SCL) directly regulating expression of PcG members during development. In so doing, SCL regulates PRC1 function, as shown by SCL-mediated global H2AK119 ubiquitination. Though PRC1 exhibits repressive roles during early embryonic development^41,45^, somatic cell reprogramming^54^ and haematopoietic development^55^, a function in lineage selection in germ layers has not been previously reported.

SCL activates expression of two specific PRC1 members, RYBP and PCGF5, both necessary for the function of ubiquitin ligase RING1B and full H2AK119 ubiquitination levels^42,56^. Thus, it is unsurprising that ubiquitination levels depend on SCL in blood-fated cells. Whilst our biochemical data indicate interaction between RYBP and SCL and co-localisation on a few genes, the mechanisms controlling RYBP recruitment and H2AK119ub deposition for most genes remain unclear. Interestingly, RYBP-PRC1 target genes are usually expressed at higher levels than targets of canonical PRC1 complexes^42^. This suggests that ncPRC1 may cause incomplete gene expression silencing, thus permitting low-level gene expression and allowing lineage-fated cells to retain plasticity. Thereafter, ncPRC1 complexes could be replaced by more potent complexes, causing irreversible gene silencing and loss of plasticity.

Our work also reveals a complex pattern of gene regulation by SCL, ETO2 and RYBP. Indeed, ChIP-seq shows locus-specific binding of ETO2 and RYBP on SCL’s 778 activated and repressed genes. Why would co-repressors bind to genes normally activated? One possibility is that co-repressors finely modulate gene expression levels, rather than simply repressing gene expression^57^. This transcriptional flexibility could allow for rapid changes in transcription patterns to adapt to changing environments, and underlies cellular plasticity and transcriptional priming. What mechanisms dictate whether a gene is bound by any combination of SCL/ETO2/RYBP and whether it is activated or repressed remain important questions. Detailed analyses of specific gene loci are required.

Finally, our work highlights some of the relationships between histone marks, TF/co-factor binding and gene expression. Although the significance of SCL-dependent H2AK119 ubiquitination over extended genomic regions remains unclear, this observation is reminiscent of the wide “blanket” of Polycomb recruitment on the inactive X chromosome^58^, a paradigm of transcriptional repression. Based on the hierarchical model that PRC1 recruits PRC2^18^, H2AK119Ub marks may facilitate recruitment of PRC2 and maintain high levels of H3K27me3 at genes normally repressed in lowly acetylated chromatin. Genome-wide, high levels of ubiquitination also suggest that the default transcriptional state may be gene expression silencing. It is noteworthy that genes activated by SCL usually harbour multiple SCL binding sites, whilst repressed genes show much fewer binding events. Multiple SCL occupancy sites may be required to robustly recruit activating epigenetic complexes to overcome a globally repressive environment.

In conclusion, this study unveils the importance of transcriptional repression by a single tissue-specific regulator in multi-lineage primed cells and over tightly defined developmental windows during which lineage-specific gene expression programmes are initiated. Complex, gene-specific patterns of transcriptional regulation promote and repress lineage-affiliated gene expression to allow single lineage selection. These mechanisms are likely to be shared by other lineage-specific regulators in development and differentiation.

## Methods

### Single molecule RNA FISH (smRNA FISH)

smRNA FISH was performed as described^59^, with some alterations. In brief, oligonucleotide libraries (18–20 nucleotide-long) were designed using Stellaris Probe Designer to bind mainly to the ORF of each target mRNA (35 oligos for *Scl*, 29 oligos for *Mesp1* and 38 oligos for *Tbx6*) and synthesised with 3′ 3NHC6 modifications. They were pooled and labelled with Alexa NHS esters using Alexa-488 (*Mesp1*—Lifetech A20000), Alexa-594 (*Scl*—Lifetech A37572) or Alexa-647 (*Tbx6* and *Gata4*—Lifetech A37573) as described by the manufacturer. Only probes with a frequency of incorporation of >90% (calculated with Geneflow nanophotometer) were used.

100,000 EB cells were adhered to poly-l-Lysine-pre-coated coverslips (20 min, 37 °C). Cells were fixed in 4% Paraformaldehyde (PFA, 20 min, room temperature -RT^o^), washed with PBS and permeabilised with 70% EtOH (overnight (o/n), 4 °C). After rehydration in wash buffer (2× SSC, 10% Formamide), cells were incubated with probes at a final concentration of 1 ng/μl in hybridisation buffer (2× SSC, 10% Formamide, 10% Dextran Sulphate, 1 mg ml^−1^ tRNA, 2 mM RNase inhibitor (RVC complex, NEB), 0.2 mg ml^−1^ BSA) in a humidity chamber (o/n), 30 °C). Cells were then washed twice in wash buffer (30 min, 30 °C) and once in wash buffer + DAPI 0.5 μg ml^−1^ (Sigma, cat# D9542) 30 min, 30 °C. Coverslips were mounted with Prolong Gold, which was allowed to polymerise o/n at RT^o^, in the dark, before imaging with a Delta Vision widefield microscope using a 100x oil objective.

### Microscopy and data analysis

To avoid bleaching, smRNA FISH images were focused and taken based on DAPI stain only. For each image, a Z-stack of 53 images was acquired for all channels (DAPI, Alexa-488, Alexa-594 and Alexa-647). Image volumes were analysed using a Fiji/ImageJ macro script that automated the analysis (available upon request). Firstly, image volumes were Z-maximum-projected through a sub-range of the original slices; the sub-range was pre-chosen to best encapsulate the signal containing range of the cells within a dataset. Next, background fluorescence in the DAPI channel was removed from images by smoothing a copy of the maximum-projected image with a coarse Gaussian filter (sigma = 80) and subtracting this from the unsmoothed image. The filtered image was then thresholded to extract the nuclear regions. From this binary image, a Voronoi filter was applied to evenly split the image into regions based on the location of the nuclear centres. Each region was then assigned to the nucleus it encapsulated. Within each nuclei-associated region, the corresponding cell auto-fluorescence in the 488 nm channel was used to identify the cell. Segmentation was performed by thresholding the cellular fluorescence using an Otsu algorithm to convert the image into a binary representation, and then, using the “Watershed” algorithm, to split any larger objects. These cellular regions were then dissected using the Voronoi regions to split them up based on the distribution of nuclear centres. Regions of fluorescence within the thresholded auto-fluorescence channel and containing a corresponding DAPI signal were retained for further analysis; all other areas were discarded. This methodology provided an accurate means of obtaining cell regions even if more than one cell were touching and also removed artifactual stained non-cellular areas. Finally, within each cell, the channels containing the foci (*Mesp1*-A448, *Scl*-A594 or *Tbx6*-A647) were processed sequentially. All channels were smoothed with a Gaussian filter (sigma = 0.5) to reduce the impact of signal noise. Following this processing step, the signal intensities were identified and measured. The foci locations were identified using the ImageJ “Find Maxima” function with a simple filtering step to remove maxima that were too large to be foci (>40 total pixel area). To avoid false positives, a specific point of cut off for positivity of 6 foci per cell was chosen after performing smRNA FISH on cells derived from ES cells knocked-out for the mRNAs of interest. Within these cells, the number of foci and also the average intensity level was measured from regions (5 × 5 pixel) centred on each foci. In addition, as a comparison, the intensity was also measured in regions surrounding the foci to give an idea of the background noise in these samples. These regions were selected by taking the coordinates of each true foci and then randomly perturbing the coordinates to find a region nearby (within 10 pixels) which did not overlap with any of the true foci locations. Data were exported to Excel for subsequent analysis and statistical testing.

Images shown in Fig. 1 and Supplementary Fig. 2 are maximum Z-stack projections of fluorescence images spanning the extent of the cells, in inverted black/white for *Scl, Mesp1* and *Tbx6*. Within each subfigure, images from the same channel were adjusted equally, for direct comparison. Before compilation of experiments, a Stratified Kruskal–Wallis test was performed to check for significant differences, and none were found. To test for significant increases or decreases in cellular sub-types, a two-tailed Fisher’s exact test was applied.

### Embryonic stem (ES) cell culture and differentiation

Mouse ES cells (J1 wild-type, *Scl*^*-/-*8^, *Scl:mCherry*, *Rybp*^*fl/fl*^*;Cre-ERT2*—generous gift from M. Vidal^45^) were maintained in DMEM high glucose supplemented with 15% batch-selected fetal bovine serum (FBS), 2 mM glutamine (Gibco/BRL), 2% LIF-conditioned medium and 1.5 × 10^−4^ M MTG. Twenty-four hours prior to differentiation, cells were passaged in LIF-containing IMDM medium. On the day of differentiation, 50,000–80,000 cells were seeded in 100 mm Petri Grade dishes in IMDM medium supplemented with 15% batch-selected FBS, 2 mM glutamine, 300 μg/ml transferrin (Roche, cat# 10652202001), 4.7 × 10^−4^ M MTG (1-Thiolglycerol, Sigma, M6145), and 50 μg ml^−1^ ascorbic acid. Cultures were maintained in a humidified incubator in 5% CO_2_/air at 37 °C.

### *Scl:mCherry* cell lines

gRNAs (sequences are shown in Supplementary Table 1) were inserted into GFP-expressing pX458 or a derivative of pX330 (Addgene), by BbsI digestion and ligation. ES cells were transfected with gRNAs and a homology donor (sequences available upon request) using Nucleofector 2b (Lonza) with programme A-013. 48hrs after transfection, single GFP-positive cells were sorted, clones expanded and checked for insertion by Sanger sequencing. The heterozygote *Scl:mCherry* cell line was generated from WT J1 cells using mCherry_gRNAs, and harbours the mCherry insertion on one allele. The *Scl*^*∆/∆*^*:mCherry* cell line was engineered from the *Scl:mCherry* cell line with mCherry∆gRNA1 and mCherry∆gRNA2, and is homozygous for deletion of the bHLH domain.

### siRNA and inhibitors

*siRNAs*—Day 2 WT EBs were transfected with 50 nM siRNA against *Eto2* (Darmacon, siGenome mouse Cbfa2t3 SMART pool, M-042769) or *Rybp* (Darmacon, siGenome mouse Rybp SMART pool, M-042769) or with siRNA control (Darmacon, siGenome control pool, non-targeting, D-001206) using lipofectamine RNAimax (LifeTechnologies, cat#13778) following the manufacturer’s recommendations. At day 3.5, transfected cells were dissociated and replated in cardiac or blast colony assay. Gene expression was assessed by qRT-PCR at days 3.5 and 4.5.

Increasing concentrations PRC1 and PRC2 inhibitors were added at day 2 of EB differentiation. Day 3.5 EBs were dissociated for downstream functional assays or gene expression analysis. PRC1 inhibitor: PRT4165 (Tocris, cat# 5047), PRC2 inhibitor: UNC1999 (Tocris, cat# 4904) or the analogue UNC2400 (Tocris, cat# 4905).

### Hydroxytamoxifen treatment of *Rybp*^*fl/fl*^*;Cre-ERT2* ES cells

Cells were treated at day 2 of EB differentiation with 0.8 μM 4-hydroxytamoxifen (4-OHT) (Sigma, H7904). Fresh media was added after 18–24 h. 4-OHT-treated EBs were allowed to develop until day 3.5 when downstream assays were carried out.

### Blast colony assay

5 × 10^4^ to 2 × 10^5^ day 3.5 EB cells were seeded in 1% methylcellulose IMDM medium, supplemented with 300 μg ml^−1^ transferrin (Roche, cat# 10652202001), 25 μg ml^−1^ ascorbic acid (Sigma, A-4544), 4 × 10^−4^ M MTG (1-Thiolglycerol, Sigma, M6145), 25% D4T (endothelial cell) conditioned media, 5 ng/ml mouse VEGF, 5 ng ml^−1^ mouse interleukin 6 and 100 ng ml^−1^ mouse cKIT. Dishes were incubated in a humidified 5% CO_2_/air incubator and colonies scored at day 4^60^. All cytokines were purchased from Preprotech.

### Cardiac assay

Day 3.5 EB cells were replated at a density of 80,000 cells per gelatin-coated 96-well plate, in StemPro34 serum-free medium supplemented with 100 U ml^−1^ penicillin, 100 μg ml^−1^ streptomycin (Gico/BRL), 2 mM glutamine (Gibco/BRL), 10 ng ml^−1^ human basic FGF, 12.5 ng ml^−1^ human FGF10, 5 ng ml^−1^ mouse VEGF and 1 mM ascorbic acid (Sigma, A-4544)^27^. All growth factors were purchased from R&D Systems. Medium was changed every other day until day 7, when cultures were analysed.

### Chondrogenic assay

After dissociation, single cell suspensions were seeded in chondrogenic medium (alpha-MEM supplemented with 10% FBS, 1% penicillin/streptomycin, 0.1 μM dexamethasone and 0.17 mM ascorbic-acid 2-phosphate) with 10 ng ml^−1^ TGFβ3 for 6 days. On day 7, the medium was replaced with 10 ng ml^−1^ BMP2. The culture was kept for another two weeks with a medium change every 3 days. On day 21, alcian blue staining was performed. Cells were fixed with 4% PFA for 30 min, rinsed twice in PBS and once with water. Cultured cells were incubated in 0.05% alcian blue solution, pH 1.5 (o/n, RT°)^26^. Presence of blue stained nodules were inspected by microscopy and cultures imaged on a microscope (BX60; Olympus) with a Q Imaging camera and OpenLab (Improvision) software.

### Antibodies

References and conditions of use of all antibodies employed in this study are shown in Supplementary Table 2.

### Immunofluorescence

Cells were fixed in 4% PFA for 10 mins on ice, washed three times in PBS (5 mins, RT°). They were then blocked and permeabilised in 0.2% Triton X-100/PBS containing 10% donkey serum (Sigma, D9663), 1 h at RT°. Cells were incubated with primary antibody in 0.2% Triton X-100/PBS containing 1% donkey serum (o/n, 4 °C). After three washes of 5 min at RT°, cells were incubated 1 h at RT° with donkey-raised secondary antibody in 0.2% Triton X-100/PBS containing 1% donkey serum and 0.5 μg ml^−1^ DAPI. Cells were washed 3× in PBS for 5 min and visualised on Zeiss 880 inverted confocal microscope. Primary antibodies: cTNT (Abcam, 1C11, 10 μg ml^−1^), CD31 (R&D systems, AF3628, 2 μg ml^−1^) and ColIIa (DSHB, II-II6B3, 5 μg ml^−1^). Secondary antibodies: Alexa-488 donkey anti-mouse (A21202, 6 μg ml^−1^), Alex-555 donkey anti-goat (Thermofisher A-21432; 5 μg ml^−1^).

### Mice

All animal work was carried out under appropriate project licences according to UK Home Office regulations and approved by the Oxford University Committee on Animal Care and Ethical Review. E9.5 embryos were harvested and dissected to collect yolk sacs. Tissues were incubated in 0.125% collagenase (Sigma, C0130) solution to achieve single cell suspensions (15 min, 37 °C). Each individual yolk sac was replated in cardiac or chondrogenic medium on an OP9 layer.

### Quantitative RT-PCR

Total RNA was extracted with Qiagen kits and DNase-treated on column. 500 ng to 1 μg of total RNA was converted into cDNA using Omniscript (Qiagen). For quantitative real-time expression analysis, qPCR reactions were performed using TaqMan universal PCR mastermix (Life Technologies, cat# 4304437) and TaqMan gene expression assays (Life Technologies). For *Gapdh* and *Gata1* genes, primers/probes were designed using Primer Express v3 software and purchased from Eurogentec. References for all primers and probes used in Taqman assays are shown in Supplementary Table 3. Expression levels were calculated relative to a control sequence in the *Gapdh* gene. All reactions were performed in duplicate using Sequence Detection System 7000 Thermocycler (Applied Biosystems).

### Immunoprecipitation (IP)

EBs were dissociated and resuspended in Buffer A (10 mM HEPES pH 7.9, 1.5 mM MgCl_2_, 10 mM KCl, and proteinase inhibitors), kept on ice for 10 min, and centrifuged for 10 s at 13,500 rpm. The nuclear pellet was resuspended in 0.5% TritonX-100/RSB100 (10 mM Tris pH 8.0, 100 mM NaCl, 2.5 mM MgCl_2_, proteinase inhibitors), vortexed on/off for 30 min, kept on ice in between. Extracts were rotated to release chromatin-bound SCL (5U Benzonase/1 μl extract for 1 h, 4 °C). Samples were centrifuged (4000 rpm, 15 min, 4 °C) and the supernatant was treated with RNase A (100 μg ml^−1^, 2–3 min) before centrifugation (13,500 rpm, 1 min) to prevent RNA-mediated carryover. The supernatant contained the soluble nuclear extracts. For IPs, nuclear extracts were precleared with Protein G dynabeads for 30 min, before incubation with antibody pre-bound to Protein G dynabeads for 3 h (SCL) or o/n (RING1B and RYBP). Beads were washed 4× in 0.5% TritonX-100/RSB100 and eluted with 1xLDS (10 min, 70 °C).

### Western blotting

Samples were run on a 4–12% precast Bis-tris gel, and transferred to a activated PVDF membrane (IPVH00010, Merck). The membrane was blocked in 5% skimmed milk/PBS, and incubated in primary antibody 1 h in 5% skimmed milk/PBS, washed four times in 0.05% tween/PBS, incubated in secondary antibody 1 h in 5% skimmed milk/PBS, washed as before and developed with the ECL prime western blotting detection kit (RPN2232, GE healthcare Life sciences). For histones, 10^6^ cells were lysed in 20 mM Tris, pH 7.4, 20 mM EDTA, pH 8, 2% SDS and 20% glycerol, boiled for 5 min and sonicated on Bioruptor Plus. Semi-quantitation of the protein bands was performed using the Biorad Chemi doc MP imaging system.

Uncropped scans are shown in Supplementary Figs. 8 and 9.

### Flow cytometry and cell sorting

Cells were stained with PE-conjugated anti-FLK1 and APC-conjugated anti-PDGFRα antibodies (eBioscience cat# 12-5821-83 and 17-1401-81) for 20 min at 4 °C, washed and sorted on BD FACS Aria II (Becton Dickinson) or Sony SH800 FACS sorter.

### SCL intracellular FACS

Cells were stained with live/dead stain kit (Thermofisher, L34955) for 20 min, fixed in 2% PFA and permeabilised in 0.1% Triton X-100. Cells were stained with anti-SCL antibody (Santa Cruz, clone C21) conjugated to Alexa-488 (Zenon Alexafluor-488 goat IgG labelling kit, Invitrogen, cat# Z25602) following manufacturer’s instructions.

### cTNT intracellular FACS

Cardiac cultures were dissociated using 0.25% trypsin/EDTA (Gibco/BRL) for 5 min at 37 °C to quickly lift the monolayer cells off the plate, then a single cell suspension was achieved with a solution of collagenase/DNase (PBS with 20% serum, 10 mg ml^−1^ collagenase A (Roche, cat#11088785103), 10 mg ml^−1^ collagenase B and 10 μg ml^−1^ DNase I (Roche, cat# 1184932001), 5–10 min at 37 °C. Cells were stained with live/dead stain (Thermofisher, cat# L34955) for 20 min, fixed in 2% PFA and permeabilised in 0.5% saponin (Sigma, cat# S7900). After a wash in PBS/0.1% saponin, cells were stained with anti-cTNT (Abcam, clone 1C11) primary antibody and Alexa-488 donkey anti-mouse (Thermofisher, cat# A21202) secondary antibody.

### Isolation of FLK1^+^ cells

Day 3.5/4 EB cells were stained with PE-conjugated FLK1 antibody (eBioscience) for 20 min. After a wash in PBS/FCS, cells were incubated with anti-PE microbeads (Miltenyi) for 15 min. FLK1^+^ cells were isolated on Myltenyi magnetic columns.

### Single-cell RNA sequencing analysis

Single cell RNA-seq read-count tables obtained from mouse embryos at different stages of development (days E6.5 to E7.75) were downloaded from http://gastrulation.stemcells.cam.ac.uk/scialdone2016^14^. The data passed the quality control and cell filtering processes described in the ref. ^14^. The R-script for the t-distributed stochastic neighbour embedding (t-SNE) analysis of different cell stages was kindly provided by A. Scialdone, and the ‘Rtsne’ function from the Rtsne package with the input from highly variable genes was used (see Method section in the ref. ^14^). To match day 3.5 EB cells to the distinct cell populations identified at different stages of mouse embryonic development, we selected 18 embryonic development-related genes highly expressed in day 3.5 EBs and superimposed the average gene expression of these genes (gene score) to visualise the enrichment on the t-SNE plot.

### RNAseq

WT and *Scl*^*−/−*^ day 3.5 FLK1^+^ EB cells were isolated and total RNA extracted. Library generation and sequencing were performed at Oxford Genomics Centre (Wellcome Trust Centre of Human Genetics). Fastq reads were aligned to the mm9 reference genome using tophat version 2.0.8b^61^. Duplicate read pairs were removed, and reads filtered for mapq > 15 using SAMtools version 0.1.19^61^. Data normalisation and differential expression analysis was carried out using edgeR^62^. Data normalisation to account for differences in sequencing depth and RNA composition was performed using the TMM method (trimmed mean of *M*-values^63^), provided by the edgeR package. Gene lists were analysed for biological processes using PANTHER overrepresentation test^64^.

### Chromatin immunoprecipitation (ChIP)

For SCL, ETO2 and RYBP ChIP, 2–5 × 10^7^ day 3.5 (SCL) and day 4 (ETO2/RYBP) FLK1^+^ cells were fixed 20 min, RT° with 2 mM EGS (ethylene glycol bis succinimidylsuccinate, ThermoFisher Scientific, cat# 10350924) followed by 1% Formaldehyde (Sigma, 252549) incubation for 10 min under gentle agitation before quenching with 0.125 M glycine. Cells were rinsed twice with PBS, lysed, and chromatin sonicated to an average size of 300 base pairs using Bioruptor Plus (Diagenode) at Power 3.0 for 17 cycles (30 s ON, 30 s OFF). The resulting chromatin was diluted 10× in ChIP dilution buffer and 1% put aside as input sample. After pre-clearing with protein A/G dynabeads (ThermoFisher Scientific), supernatants were incubated with appropriate antibodies o/n. Beads were washed in low salt/high salt LiCl buffers and twice with Tris/EDTA. Complexes were eluted from beads in a thermomixer (65 °C, 15 min) and eluates and reserved inputs reverse cross-linked (65 °C, o/n). Samples were treated with RNaseA, proteinase K and phenol/chloroform-purified.

### ChIP-sequencing

ChIP samples were submitted to library preparation using NEBNext Ultra DNA Library prep kit and multiplexing barcodes (NEB, E7370) following Illumina’s instructions. Samples were quantified by the KAPA Library Quantification Kit (Roche diagnostics, cat# 7960140001) and their sizes verified on a Bioanalyser 2100 (Agilent). Libraries were sequenced using NextSeq v2 kit (75 cycles, Illumina, FC-404-2005) on a NextSeq 550 system with paired-end reads for 37 cycles. Sequences were de-multiplexed, aligned to the Mus musculus (mm9) genome using Bowtie (version 1.1.2), and filtered to remove duplicate mapped reads. Peak calling was performed with MACS2 (version 2.0.10) using default parameters for SCL and ETO2 ChIP; the broad option was applied for RYBP ChIP. MIG (Multi-Image Genome viewer) was used to visualise the data, filter and identify peaks^65^. Data for heatmaps and meta gene plots were generated using HOMER annotatePeaks command and visualised with Java_Treeview. GO analysis of genomic regions was performed with Stanford GREAT online tool^66^. The ATAC-seq procedure and data will be reported somewhere else.

### Histone ChIP-sequencing-Rx

Histone ChIPSeq-Rx was carried out according to published protocols^37^. Briefly, day 4 mCherry-positive sorted cells were mixed in a 2:1 ratio to Drosophila S2 cells. Samples were prepared and sequencing was performed as above. Data were mapped using an in-house pipeline. Reads were aligned to Mus musculus (mm9) and Drosophila melanogaster (dm3) genomes. The number of reads mapping to the genomes as well as the derived normalisation factor for each histone mark are provided in Supplementary Data 4. Peak calling was performed using MACS2 (version 2.0.10) using parameters for broad peak calling and respective inputs as reference. Tag directories were created using Homer and the generated BedGraphs were visualised on UCSC Genome Browser as custom tracks, after scaling the tag densities by the normalisation factor for each histone mark. Histograms and heatmaps were generated using AnnotatePeaks.pl command with up to −100 kb to 100 kb around the indicated genomic regions separated into 25 equally sized bin; the ChIP fragment coverage was scaled by the derived normalisation factor.

### Code availability

All codes used in this study can be accessed from the GEO database (accession number GSE104883).

## Supplementary information

Supplementary Information

Description of Additional Supplementary Files

Supplementary Data 1

Supplementary Data 2

Supplementary Data 3

Supplementary Data 4

Reporting Summary

## Data Availability

All ChIP-seq and RNA-seq datasets are deposited at the NCBI GEO database under the accession number GSE104883. All other data supporting the results of this study are available within the article and its supplementary information files and from the corresponding author upon request. A reporting summary is available as a Supplementary Information file.
