# Supplementary material for: SCL/TAL1 cooperates with Polycomb RYBP-PRC1 to suppress alternative lineages in blood-fated cells
Source: Nat Commun. 2018 Dec 18;9:5375. doi: 10.1038/s41467-018-07787-6 (PMC6299140; doi:10.1038/s41467-018-07787-6)
Supplement: Supplementary file 1 — Supplementary Information [file 41467_2018_7787_MOESM1_ESM.pdf]

Supplementary information

SCL/TAL1 cooperates with Polycomb RYBP-PRC1 to suppress alternative lineages in blood-fated cells

Chagraoui *et al.*

Supplementary Figure 1

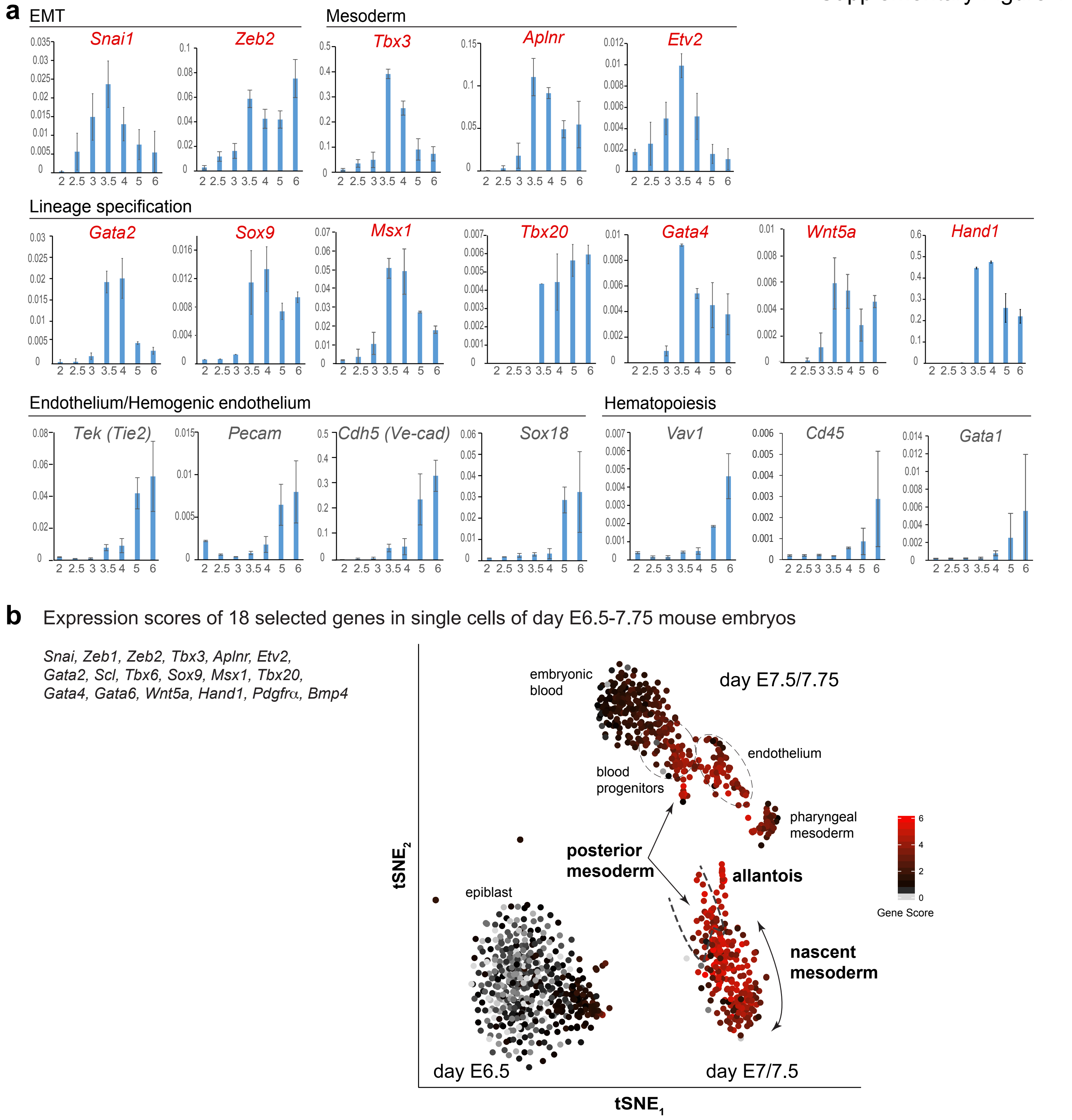

Supplementary Figure 1. Day 3.5 EBs correspond to day E7/7.5 mouse embryos, related to Figure 1.

(a) Gene expression analyses by RT-qPCR throughout a time-course of EB differentiation (day 2 to day 6).
To further define the embryonic developmental stage corresponding to day 3.5 EBs, we tested expression of more markers of key developmental time points and cell types. Expression of genes that mark epithelial to mesenchymal transition (EMT, *Snai1, Zeb2*) and mesoderm (*Tbx3, Aplnr, Etv2*) starts at days 2.5/3 and peaks at day 3.5. Confirming the data on *Scl, Mesp1* and *Tbx6* (Fig. 1a), expression of markers of lineage-fated progenitors arising from mesoderm becomes robust at day 3.5 (hematovascular *Gata2*; chondrogenic *Sox9*; muscle *Msx1*; and cardiac *Tbx20, Gata4, Wnt5a, Hand1*). In contrast, endothelial markers, also associated to the hemogenic endothelium, are lowly expressed at days 3.5/4 and increase considerably at days 5/6 (*Tek* (*Tie2*), *Pecam, Cdh5* (*Ve-cadherin*), *Sox18*). Finally, the markers of more mature hematopoietic cells (*Vav1, Gata1, Cd45*) increase above background at day 5 and are expressed at higher levels at day 6. Therefore, day 3.5 sees strong expression of genes associated with cell fate specification in the primitive streak (EMT, mesoderm patterning and lineage specification). In red, markers that are maximally expressed at days 3.5/4 and have been used in (b).
Mean of two independent experiments ± SD.
(b) tSNE plot of 1,205 single cells (published data from Scialdone et al.[1]) showing the average gene expression score of 18 genes highly expressed in our day 3.5 EBs. Whilst some of the selected genes are expressed in endothelium and blood progenitors, the highest gene expression scores are observed in cells corresponding to nascent and posterior mesoderm as well as the allantois, thus confirming that day 3.5 EB cells most closely correspond to a primitive streak/mesoderm specification stage.

a

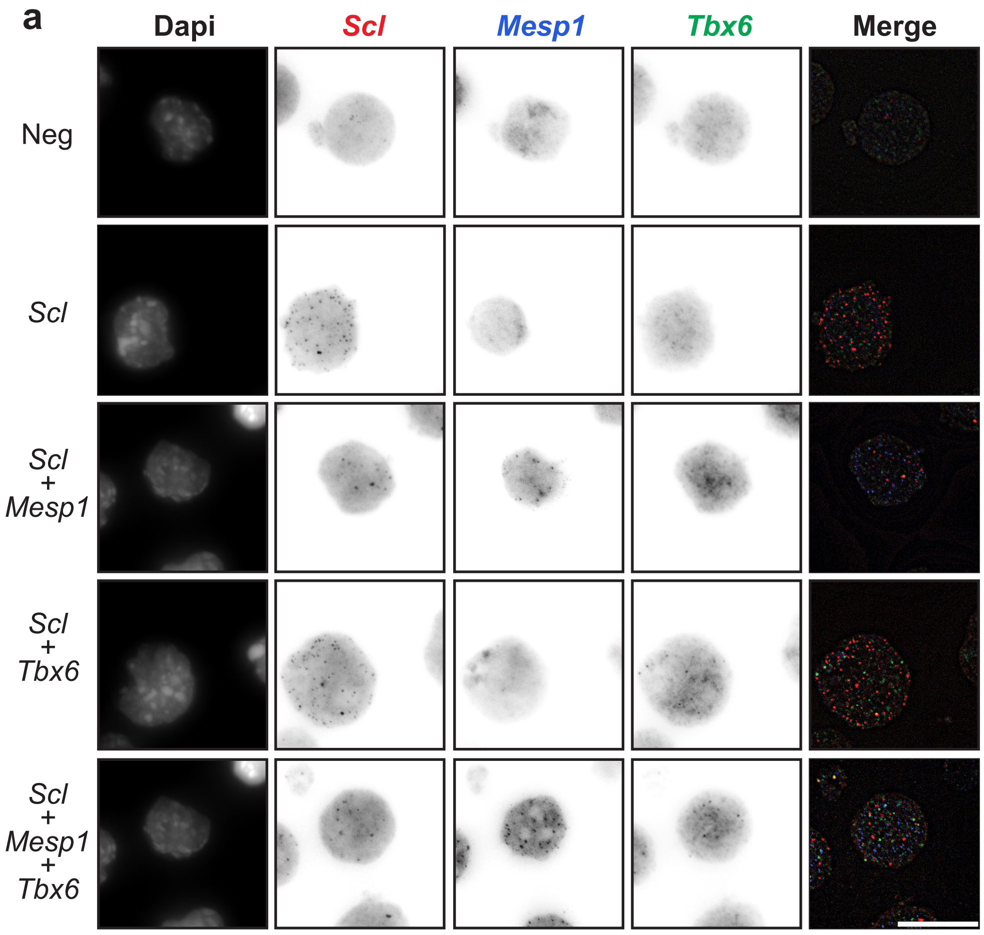

e

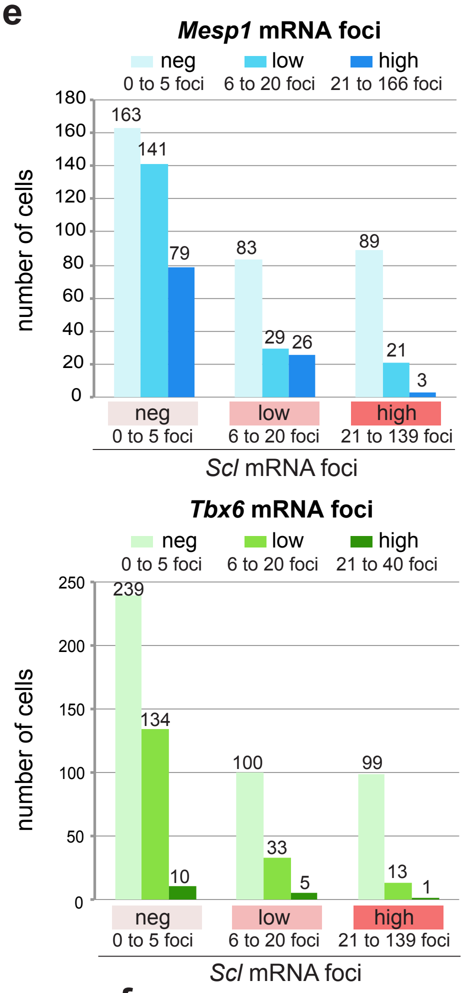

b

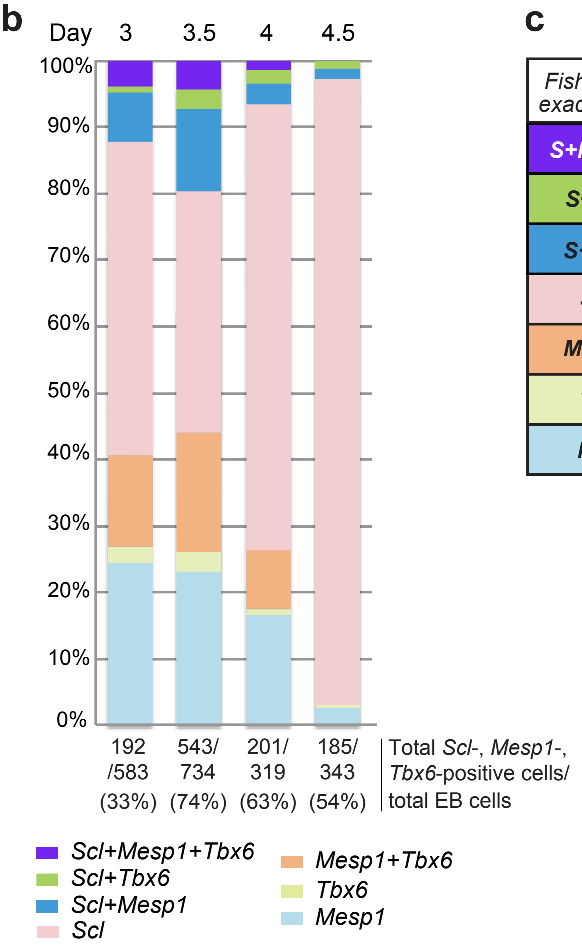

c

Fisher's exact test	*Day 3 vs day 3.5*	*Day 3.5 vs Day 4*	*Day 4 vs Day 4.5*
S+M+T	**0.0072**	**0.0221**	0.1113
S+T	**0.0061**	0.4585	0.4358
S+M	**< 0.0001**	**< 0.0001**	1
S	**< 0.0001**	**< 0.0001**	**0.0291**
M+T	**< 0.0001**	**< 0.0001**	**< 0.0001**
T	0.0628	**0.0498**	0.6113
M	**< 0.0001**	**0.0067**	**< 0.0001**

f

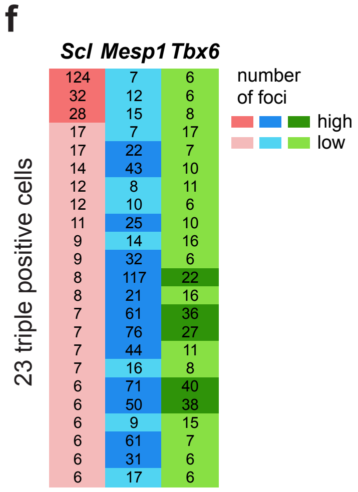

d

day 3.5	Observed	Expected
M+G	74%	56%
M	26%	44%
two-tailed test	<0.0001	

day 3.5	Observed	Expected
S+M+G	87%	47%
S+M	13%	53%
two-tailed test	<0.0001	

Supplementary Figure 2. **Single molecule mRNA FISH (smRNA FISH), related to Figure 1.**

(a) Representative images of smRNA FISH performed on day 3.5 negative, *Scl*, *Scl*+*Mesp1*, *Scl*+*Tbx6* and *Scl*+*Mesp1*+*Tbx6* positive single EB cells. Images are maximum z-stack projections of fluorescence images spanning the extent of the cell, in inverted black/white for *Scl, Mesp1* and *Tbx6*. For each subfigure, images from same channel (*Scl, Mesp1* or *Tbx6*) are adjusted equally for direct comparison. Scale bar: 11.3μm

(b) Distribution of *Scl, Mesp1* and *Tbx6* positive cells in day 3.0, 3.5, 4.0 and 4.5 EBs. The total number of EB cells and percentage of total *Scl*-, *Mesp1*- and *Tbx6*-positive cells are indicated for each timepoint. Cells are considered positive for a given marker when harbouring 6 or more foci.

(c) Fisher's exact test was performed between cell populations shown in panel B at the indicated time points. In bold, test <0.05.

(d) Co-expression analysis of *Scl, Mesp1* (S, M) and another cardiac-defining gene, *Gata4* (G), by smRNA FISH, at day 3.5. Observed and expected frequencies, two-tailed exact probability (Fisher's exact test).

(e) Distribution of day 3.5 EB cells based on number of *Scl, Mesp1* and *Tbx6* foci. Neg, negative: 0 to 5 foci; low, 6 to 20 foci; high, 21 to highest number of foci detected for each marker. Top and bottom: distribution of cells harbouring no, low and high numbers of *Mesp1* (top) or *Tbx6* (bottom) transcripts within each *Scl* fraction. Note the low frequency of cells harbouring high numbers of both *Scl* and *Mesp1* foci (3 cells) versus those harbouring low numbers of *Scl* foci and high numbers of *Mesp1* foci (26 cells). A similar pattern is observed between *Scl* and *Tbx6* (1 cell versus 5 cells).

(f) Number of foci for each marker in the 23 triple positive cells detected in day 3.5 EBs.
Cells containing high versus low foci numbers are color-coded as in **(d)**. Note the absence of cells co-expressing high levels of the three markers.

Supplementary Figure 3

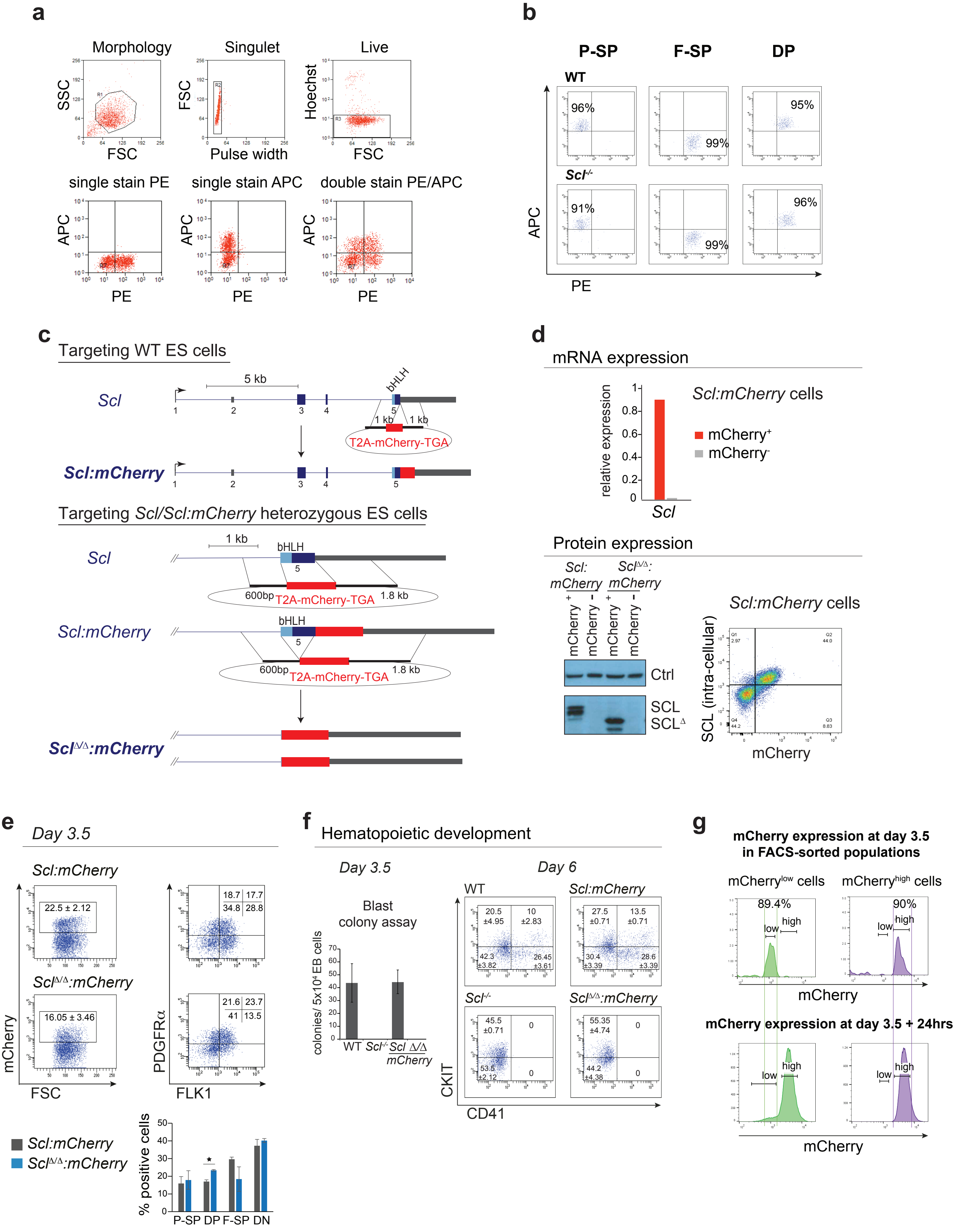

Supplementary Figure 3, related to Figures 2 and 3.

(a) Representative flow cytometry analysis of day 3.5 EB cells.

(b) Representative examples of post-sort purities after purification of FKL1- and PDGFRα-positive populations from day 3.5/4.5 EBs

(c-f) Functional validation of *Scl:mCherry* and *Scl*$^{\Delta/\Delta}$*:mCherry* reporter cell lines.

(c) Schematic representation of the targeting strategies generating the *Scl:mCherry* (top) and *Scl*$^{\Delta/\Delta}$*:mCherry* (bottom) reporter lines. Dark blue boxes; exons; light blue box, sequences coding for the bHLH domain; thin lines, introns; the T2A-mCherry-TGA sequences and the extent of homology arms are shown in circular vectors.

(d-f) Validation of the mCherry reporter lines. **(d)** Top and bottom left: in day 3.5 EBs, expression of WT *Scl* mRNA (RT-qPCR) WT SCL and truncated SCL$^{\Delta}$ proteins (Western Blotting) was exclusively detected in the mCherry^{+} cell population; bottom right, FACS analysis showed correlation between mCherry and WT SCL expression in *Scl:mCherry*$^{+}$ cells. **(e)** Day 3.5 *Scl:mCherry* and *Scl*$^{\Delta/\Delta}$*:mCherry* EBs contained similar percentages of mCherry^{+} cells (left) and FLK1-/PDGFRα-positive cells (right); bottom right, mean of 2 experiments ±SD; student's t-test, *$p<0.05$). **(f)** The mCherry reporter lines reproduced the hematopoietic phenotype of their WT and *Scl*$^{-/-}$ counterparts. The hematopoietic potential of *Scl:mCherry* cells was similar to that of WT cells in day 3.5 blast colony assays (left) and in the production of CD41^{+} blood cells at day 6 (right). The *Scl*$^{\Delta/\Delta}$*:mCherry* cells, like *Scl*$^{-/-}$ cells, showed complete absence of hematopoietic activity at days 3.5 and 6. For each assay, mean (±SD) of 2 independent experiments.

(g) mCherry^{low} and mCherry^{high} expressing cells were FACS-sorted from day 3.5 EBs to a purity of 89.4% and 90%, respectively (top). The histograms show the levels of mCherry in day 3.5 FACS-sorted cells (top) and 24hrs post-sort after re-aggregation (bottom); n=2.

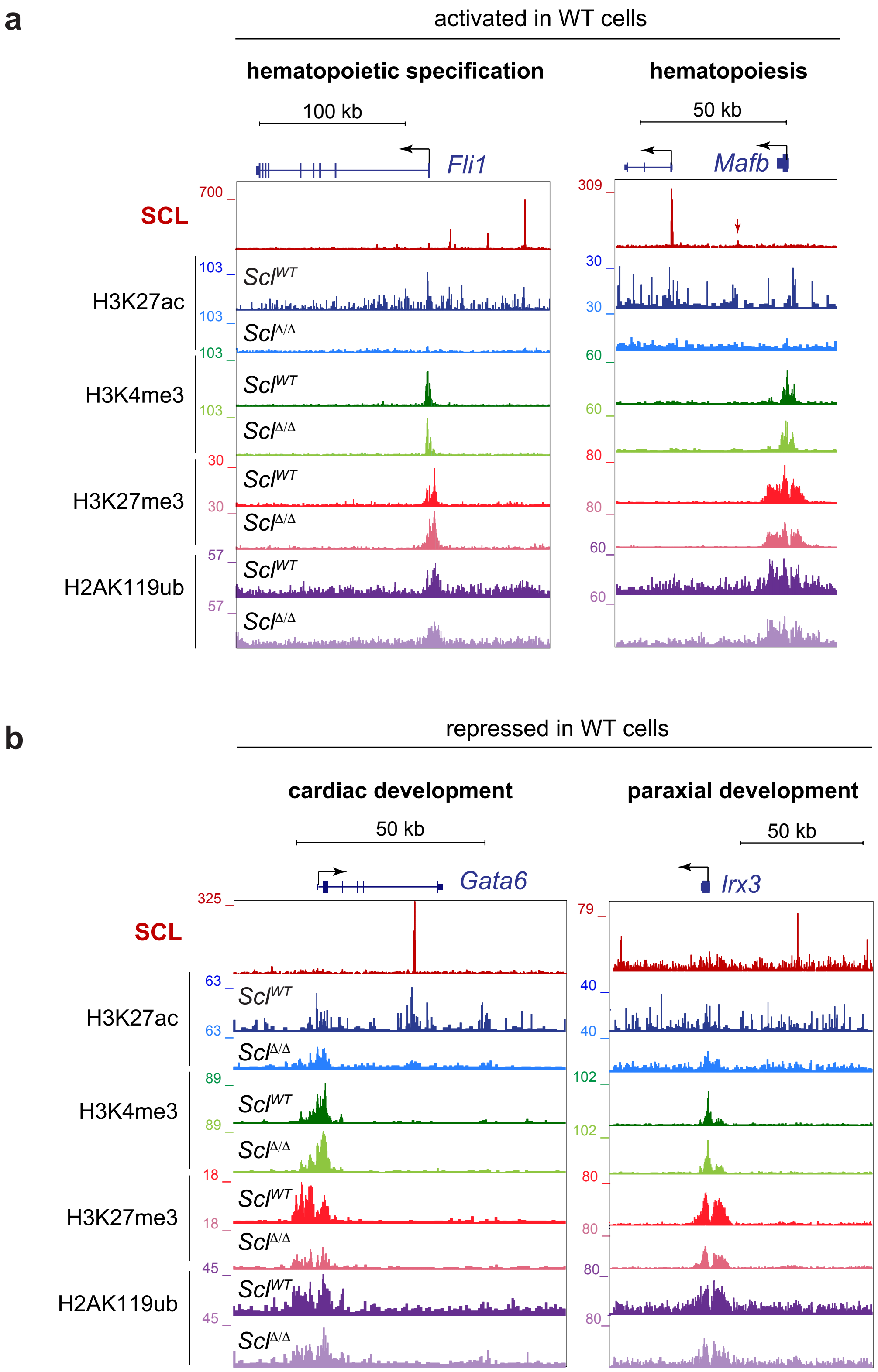

Supplementary Figure 4. UCSC tracks of selected SCL target genes, related to Figure 5

UCSC tracks showing SCL binding and histone mark distribution on **(a)** activated (*Fli1, Mafb*) and **(b)** repressed (*Gata6, Irx3*) SCL direct DEGs in day 4 Scl^{WT} and $Scl^{\Delta/\Delta}$ $mCherry^{+}$ cells.

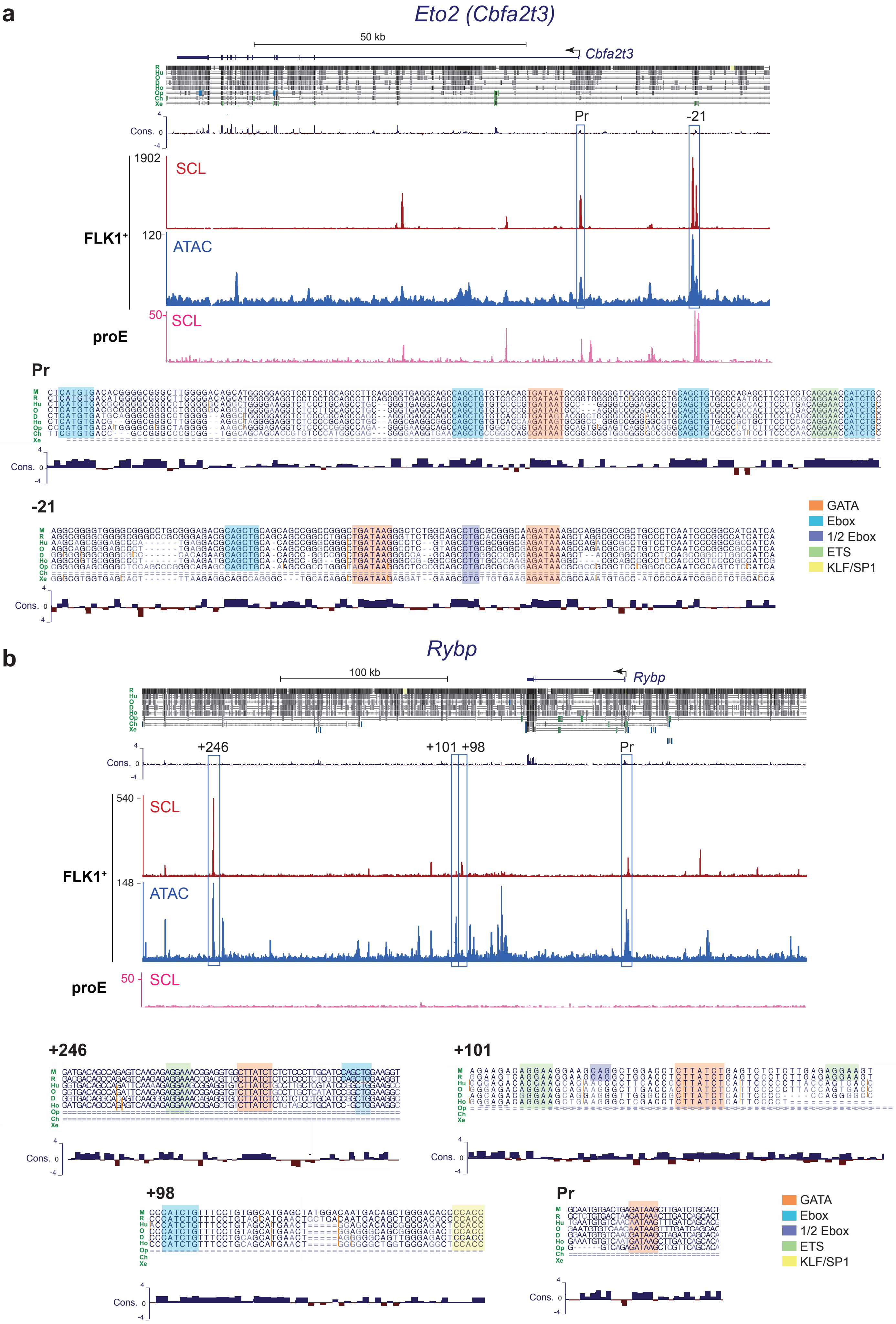
a
Eto2 (Cbfa2t3)
50 kb
Cbfa2t3
R
Hu
O
D
Ho
Op
Ch
Xe
Cons.
Pr
-21
1902
SCL
FLK1+
120
ATAC
50
SCL
proE
Pr
M
-21
GATA
Ebox
1/2 Ebox
ETS
KLF/SP1
b
Rybp
100 kb
Rybp
+246
+101 +98
Pr
540
SCL
FLK1+
148
ATAC
50
SCL
proE
+246
+101
+98
Pr
GATA
Ebox
1/2 Ebox
ETS
KLF/SP1

Supplementary Figure 5. *Eto2* (*Cbfa2t3*) and *Rybp* genomic loci, related to Figure 6

Comparative genomic analyses of **(a)** *Eto2* (*Cbfa2t3*) and **(b)** *Rybp* loci across vertebrate species. Top, Cons, *PhiloP* conservation. Tracks of SCL ChIP-seq (red) and ATAC-seq (blue) from day 3.5 FLK1$^+$ cells and SCL ChIP-seq from fetal liver pro-erythroblasts (proE, pink) are shown. *Cis*-elements are named according to their distance (in kb) to the promoter. Bottom, sequence comparison and known SCL DNA-binding motifs (GATA, WGATAR; Ebox, CANNTG; ½ Ebox, CTG; ETS, AGGAA; KLF/SP1, CCAACC) are shown for select SCL peaks (boxed).

R, rat; Hu, human; O, orang-outan; D, donkey; Ho, horse; Op, opossum; Ch, chicken; Xe, xenopus. Pr, promoter.

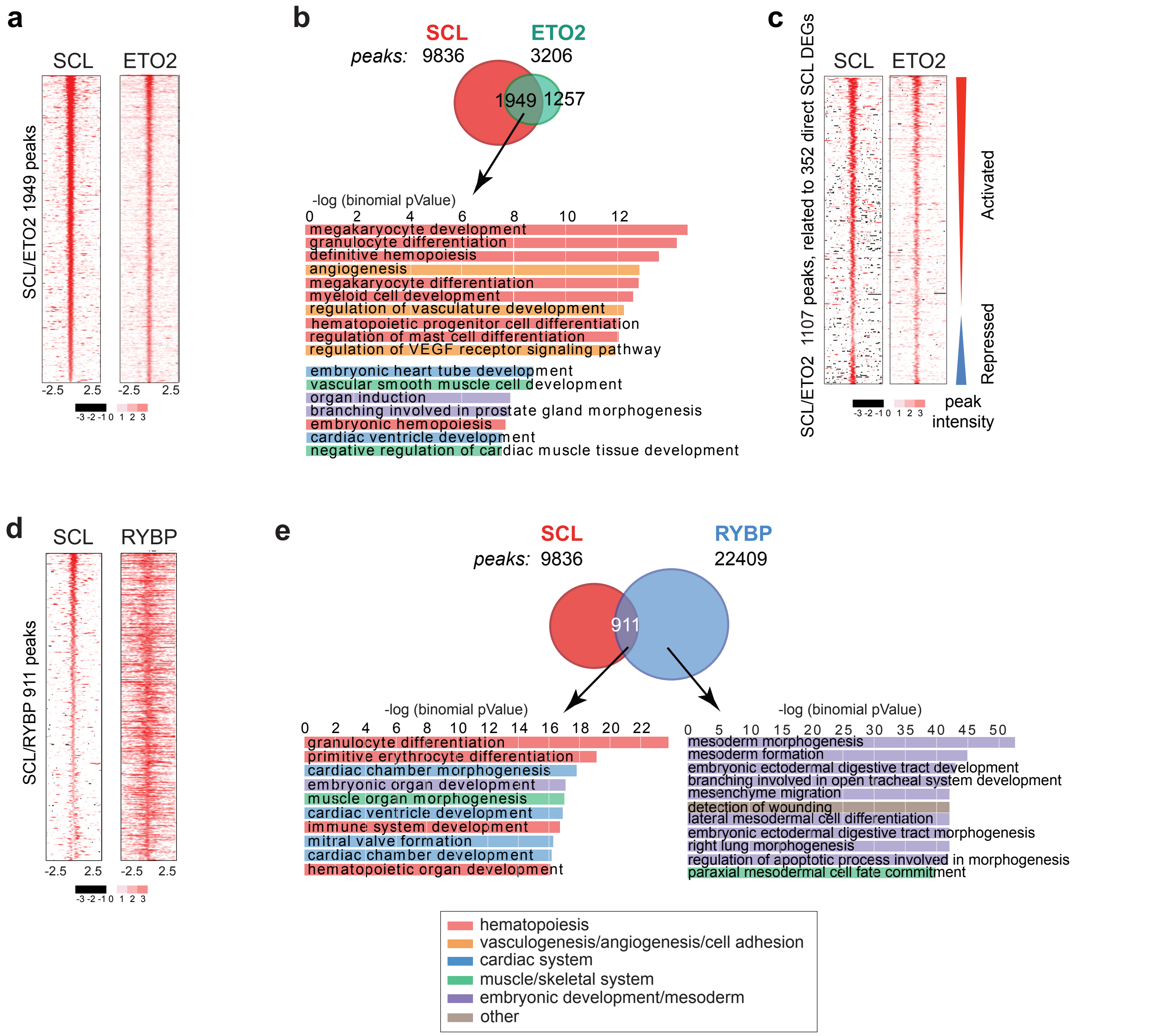

Supplementary Figure 6. GO biological terms related to ETO2 and RYBP peaks and associated genes, related to Figure 6

(a) Heatmap representation of SCL and ETO2 common 1949 ChIP-seq signals ± 2.5kb, sorted on SCL peaks.
(b) GO biological processes related to the genes associated to the 1949 common SCL/ETO2 peaks (GREAT analysis).The top 10 categories are related to hematopoiesis and vasculogenesis; categories with a lower binomial pValue are also shown that are associated to alternative mesodermal lineages and developmental processes.
(c) Heatmap representation of SCL and ETO2 ChIP-seq signals ± 2.5Kb, sorted on SCL/ETO2 common 1107 peaks associated to SCL direct activated and repressed DEGs and ranked on gene expression fold change.
(d) Heatmap representation of SCL and RYBP common 911 ChIP-seq signals ± 2.5kb, sorted on SCL peaks.
(e) GO biological processes related to the genes associated to the 911 common SCL/RYBP peaks and to RYBP-only peaks (GREAT analysis). The top 10 categories are shown.

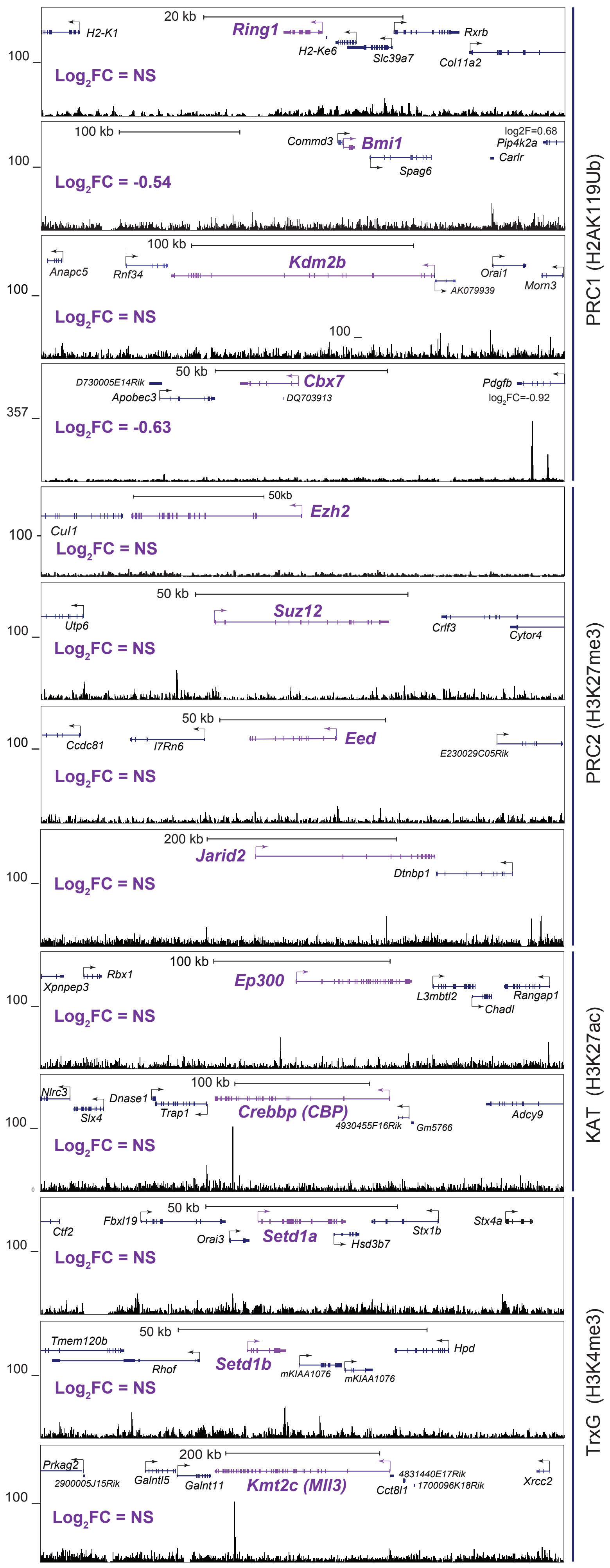

Supplementary Figure 7. SCL ChIP-seq tracks of selected genes coding for chromatin regulators, related to Figure 6.

The regulatory complex the genes belong to and the associated chromatin mark (in brackets) are indicated on the side. Log_2FC represents the fold change in gene expression between day 3.5 *Scl*$^{-/-}$ and WT FLK1+ cells (RNA-seq data). NS, non significant.
None of the *Pcgf* genes (except *Pcgf5*, Fig. 6f, 6g) are targets of SCL (not shown).

a *Related to Fig. 5g (antibody titration)*

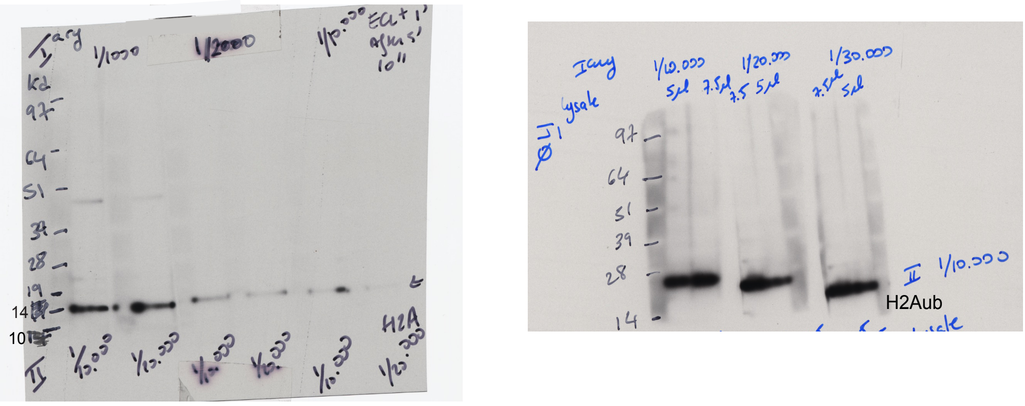

b *Related to Fig. 7c top*

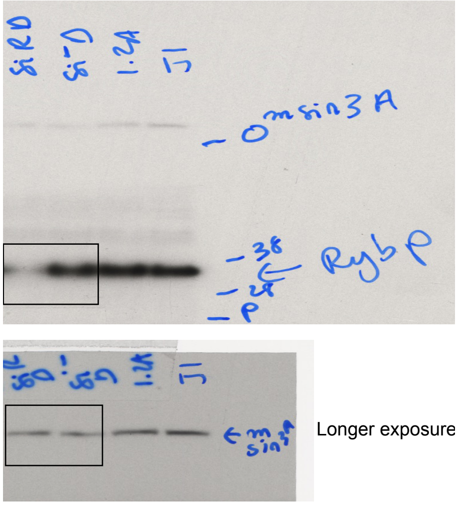

c *Related to Fig. 7c bottom*

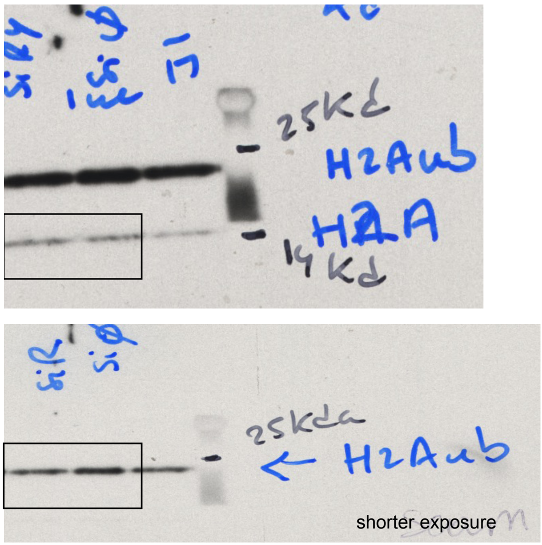

d *Related to Fig. 7i*

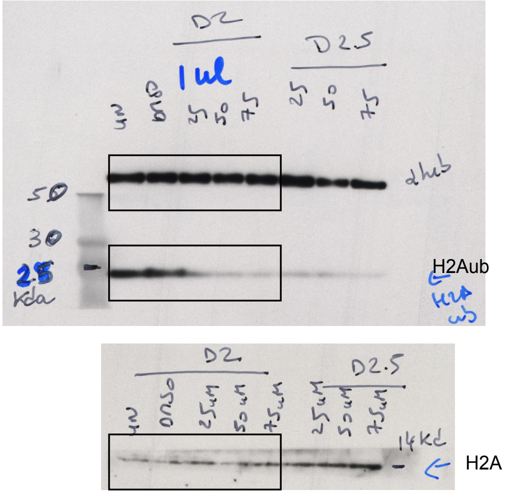

e *Related to Fig. 7k*

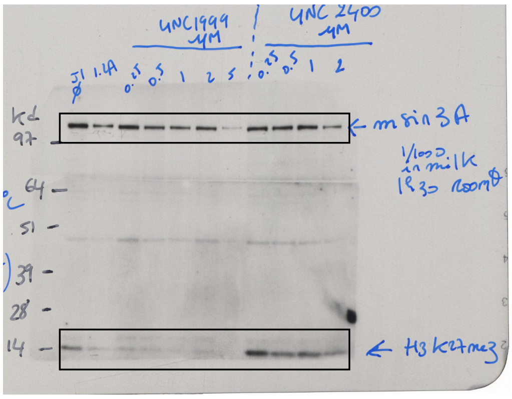

J1: WT ES cells
1.2A: SCL-null ES cells
Si⁻ D, Si Ø: siRNA neg Dharmacon
Si R D: siRNA against RYBP Dharmacon

Supplementary Figure 8, related to Figures 5 and 7
Uncropped scans of Western Blots showing the specificity of the antibodies used in the study (a) or corresponding to the gels presented in Figures 5 and 7 (b-e).

a *Related to Fig. 6c and Fig. 6h top*

SCL IP

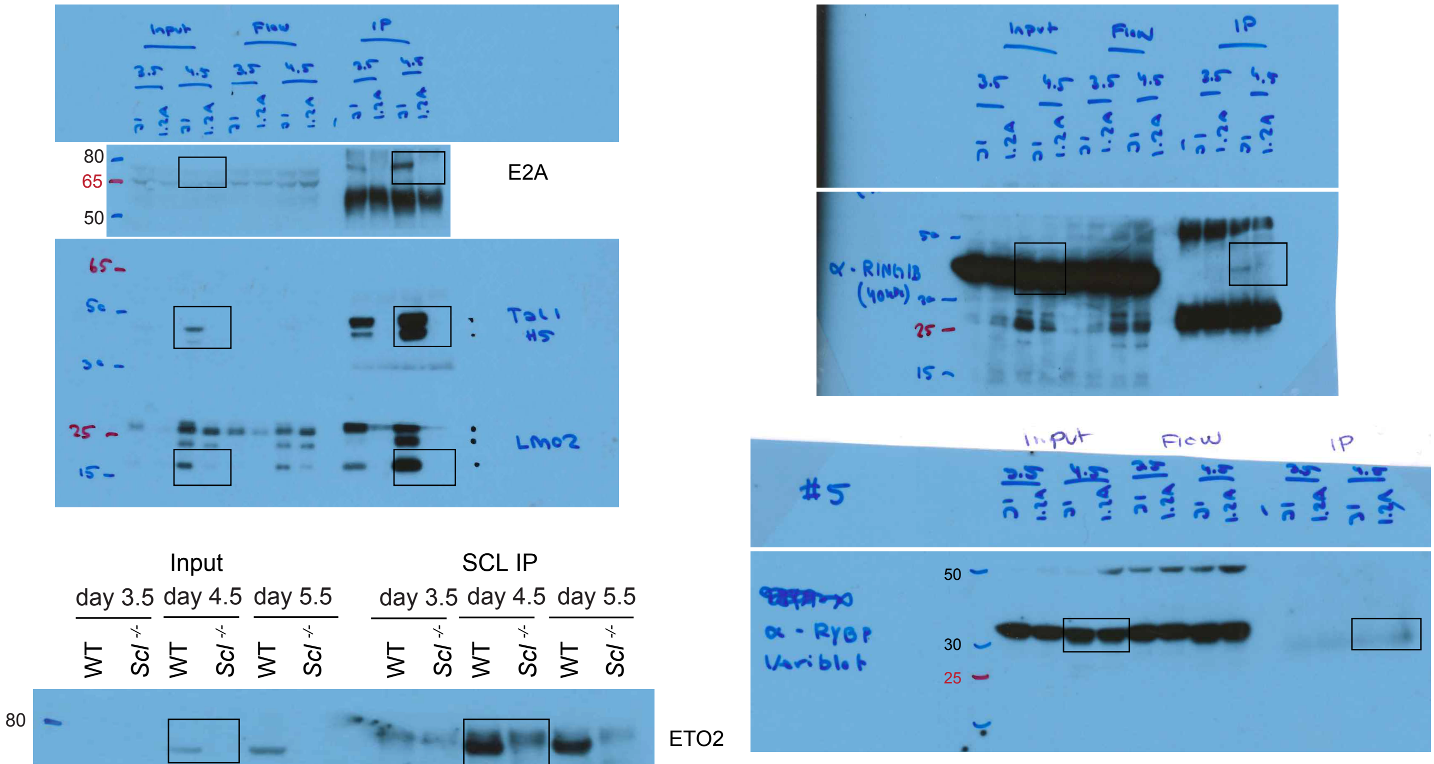

J1: WT ES cells; 1.2A: SCL-null ES cells

b *Related to Fig. 6h bottom, left*

RYBP IP

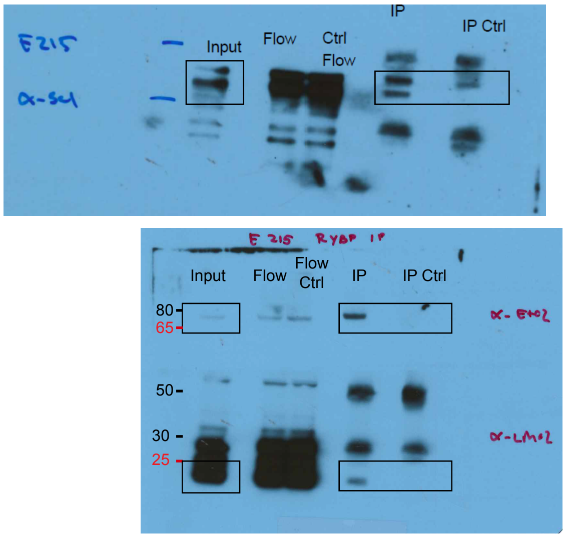

c *Related to Fig. 6h bottom, right*

RING1B IP

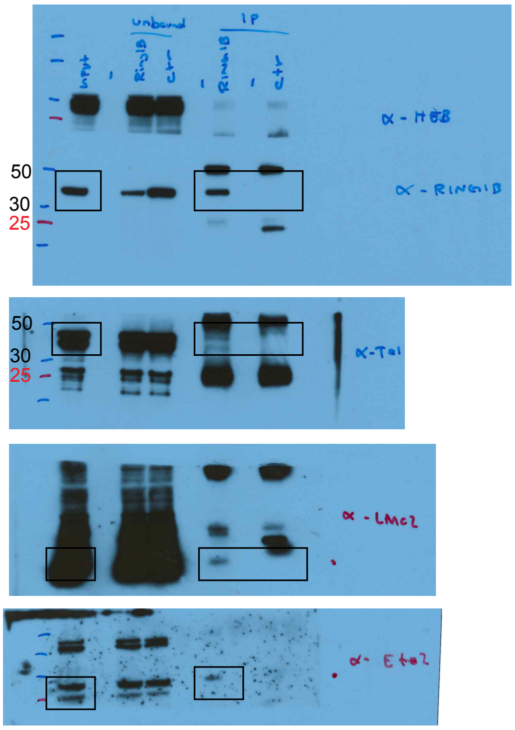

Supplementary Figure 9, related to Figure 6
Uncropped scans of Western Blots corresponding to the gels presented in Figure 6.

Supplementary Reference

1. Scialdone, A. *et al.* Resolving early mesoderm diversification through single-cell expression profiling. *Nature* **535**, 289-293 (2016).

Supplementary Tables

Supplementary Table 1. Sequences of oligonucleotides used as guide RNAs

Name	5'-3'
mCherry_gRNA_FW	CACC ggtggcccccggtgatgcgtc
mCherry_gRNA_RV	AAAC gacgcatcaccgggggccacc
mCherryΔ_gRNA1_FW	CACC ggaagatgcgccgcactactt
mCherryΔ_gRNA1_RV	AAAC aagtagtgcggcgcatcttcc
mCherryΔ_gRNA2_FW	CACC ggctcctctgtgtaactgtcc
mCherryΔ_gRNA2_RV	AAAC ggacagttacacagaggagcc

Supplementary Table 2. References and conditions of use of antibodies employed in this study

Description	***Cat. No.***	***Supplier***	***Western***	***IP***	***FACS/IF***	***ChIP-seq***
E2A	Sc-763	Santa Cruz	1:200	-	-	-
ETO2	Sc-9739	Santa Cruz	1:200	-	-	-
LMO2	Mca2744ga	AbD Serotec	1:2,000	-	-	-
RING1B	D139-3	MBL	1:2,000	1ug/	-	-
RYBP	AB3637	Millipore	1:1,000	1ug/	-	5 μg/Chip
SCL	Sc-12984	Santa Cruz	1:200	4ug/1mg extract	64 ng / N/A	-
cTNT	Ab8295 Clone 1C11	Abcam	-	-	4 / 10 μg ml^{-1}	-
CD31	AF3628	R&D systems		-	N/A / 2 μg ml^{-1}	
Alexa488 donkey anti-mouse	A21202	Thermofisher			2 / 6.6 μg ml^{-1}	
Alexa555 donkey anti-goat	A21432	Thermofisher			N/A / 5 μg ml^{-1}	
mSIN3A	Sc-994	Santa Cruz	1:1,000	-	-	-
H2A	ab18255	Abcam	1:5,000	-	-	-
H3K27me3	ABE44	Millipore	1:10,000	-	-	-
Goat anti-mouse IgG-HRP	Sc-2031	Santa Cruz	1:10,000	-	-	-
Donkey anti-rabbit IgG-HRP	Sc-2313	Santa Cruz	1:10,000	-	-	-
Donkey anti-goat IgG-HRP	Sc-2020	Santa Cruz	1:10,000	-	-	-
Easyblot anti-goat	GTX628547-01	Genetex	1:1,000	-	-	-

Easyblot anti-mouse	GTX221667-01	Genetex	1:1,000	-	-	-
Easyblot anti-rabbit	GTX221666-01	Genetex	1:1,000	-	-	
FLK1-PE	12-5821-83	eBioscience	-	-	1:100	-
CD140a-APC	17-1401-81	eBioscience	-	-	1:100	-
ETO2	Sc-9741 X	Santa Cruz	-	-	-	40 μg/Chip
SCL rabbit antiserum	-	-	-	-	-	15 μl/Chip
H3K4me3	ab8580	Abcam	-	-	-	2 μg/Chip
H3K27ac	ab 4729	Abcam	-	-	-	3 μg/Chip
H3K27me3	39155	Active motif	-	-	-	3 μg/Chip
H2AK119ub	D27C4	Cell Signaling	1: 20,000	-	-	10 μl/Chip

Supplementary Table 3. References for gene primers and probes used in qPCR Taqman assays

Gene name	Taqman assay ID (FAM-MGB)
Fgf5	Mm00438919_m1
Brachyury	Mm01318252_m1
Flk1	Mm00440109_g1
Scl	Mm01187033_m1
Mesp1	Mm00801883_g1
Tbx6	Mm01278677_m1
Tnnt2	Mm00441922_m1
Tnni3	Mm00437164_m1
Myh6	Mm00440359_m1
Sox9	Mm00448840_m1
Col2a1	Mm01309565_m1
Runx1	Mm01213404_m1
Fli1	Mm00484410_m1
Zfpm1	Mm00494336_m1
Gse1	Mm01242733_m1
Gata6	Mm00802636_m1
Eto2	Mm00486784_m1
Rybp	Mm04203868_g1
Pcgf5	Mm00512831_m1
Ring1b (Rnf2)	Mm00803321_m1
Ezh2	Mm00468464_m1
Ezh1	Mm01292499_m1
Gata2	Mm00492301_m1
Snai1	Mm00441533_g1
Zeb2	Mm00497196_m1
Tbx3	Mm01195726_m1
Alpnr	Mm00442191_s1
Etv2	Mm00468389_m1
Msx1	Mm00440330_m1
Tbx20	Mm01153088_m1

Gata4	Mm01310445_m1
Wnt5a	Mm00437347_m1
Hand1	Mm00433931_m1
Tie2	Mm00443243_m1
Pecam	Mm01242576_m1
Vecad	Mm00486938_m1
Sox18	Mm00656049_gH
Vav1	Mm01232047_m1
Cd45	Mm01293577_m1
Gata1 Forward	CAG-GAA-TTC-CCT-CCA-TAC-TGT-TGA-G
Gata1 Reverse	AGA-GAA-GCT-GAG-GCC-TAC-AGA
Gata1 probe ***(FAM-TAMRA)***	CAC-TCC-CCA-GTC-TTT-C
Gapdh forward	CAT-CCA-TGA-CAA-CTT-TGG-TAT-CGT
Gapdh reverse	CAG-TCT-TCT-GGG-TGG-CAG-TGA
Gapdh probe ***(FAM-TAMRA)***	AAG-GAC-TCA-TGA-CCA-CAG-TCC-ATG-C
