# Supplementary material for: SCL/TAL1 cooperates with Polycomb RYBP-PRC1 to suppress alternative lineages in blood-fated cells
Source: Nat Commun. 2018 Dec 18;9:5375. doi: 10.1038/s41467-018-07787-6 (PMC6299140; doi:10.1038/s41467-018-07787-6)
Supplement: Supplementary file 2 — Description of Additional Supplementary Files [file 41467_2018_7787_MOESM2_ESM.pdf]

Supplementary Data

Supplementary Data 1. SCL's 778 direct DEGs (peak location, gene names, fold change (FC) values in *Scl*$^{-/-}$ versus WT cells).
Related to Fig. 4.

Supplementary Data 2. Description of the DEGs associated to the first 100 strongest SCL ChIP-seq peaks and related to hematopoietic, endothelial and repression processes.
Related to Fig. 4.

Supplementary Data 3. Distribution of SCL's 778 direct DEGs according to SCL, ETO2 and RYBP occupancy.
Related to Fig. 6, Supplementary Fig. 6.

Supplementary Data 4. Normalisation factors for histone ChIP-seq Rx.
Related to Fig. 5
