# Supplementary material for: SCL/TAL1 cooperates with Polycomb RYBP-PRC1 to suppress alternative lineages in blood-fated cells
Source: Nat Commun. 2018 Dec 18;9:5375. doi: 10.1038/s41467-018-07787-6 (PMC6299140; doi:10.1038/s41467-018-07787-6)
Supplement: Supplementary file 7 — Reporting Summary [file 41467_2018_7787_MOESM7_ESM.pdf]

nature research

Corresponding author(s): Catherine Porcher

Reporting Summary

Nature Research wishes to improve the reproducibility of the work that we publish. This form provides structure for consistency and transparency in reporting. For further information on Nature Research policies, see Authors & Referees and the Editorial Policy Checklist.

Statistical parameters

When statistical analyses are reported, confirm that the following items are present in the relevant location (e.g. figure legend, table legend, main text, or Methods section).

n/a	Confirmed	
☐	☒	The exact sample size (*n*) for each experimental group/condition, given as a discrete number and unit of measurement
☐	☒	An indication of whether measurements were taken from distinct samples or whether the same sample was measured repeatedly
☐	☒	The statistical test(s) used AND whether they are one- or two-sided<br>*Only common tests should be described solely by name; describe more complex techniques in the Methods section.*
☒	☐	A description of all covariates tested
☒	☐	A description of any assumptions or corrections, such as tests of normality and adjustment for multiple comparisons
☐	☒	A full description of the statistics including central tendency (e.g. means) or other basic estimates (e.g. regression coefficient) AND variation (e.g. standard deviation) or associated estimates of uncertainty (e.g. confidence intervals)
☒	☐	For null hypothesis testing, the test statistic (e.g. *F*, *t*, *r*) with confidence intervals, effect sizes, degrees of freedom and *P* value noted<br>*Give P values as exact values whenever suitable.*
☒	☐	For Bayesian analysis, information on the choice of priors and Markov chain Monte Carlo settings
☒	☐	For hierarchical and complex designs, identification of the appropriate level for tests and full reporting of outcomes
☒	☐	Estimates of effect sizes (e.g. Cohen's *d*, Pearson's *r*), indicating how they were calculated
☐	☒	Clearly defined error bars<br>*State explicitly what error bars represent (e.g. SD, SE, CI)*

Our web collection on statistics for biologists may be useful.

Software and code

Policy information about availability of computer code

Data collection	The Fiji/ImageJ macro used for single molecule mRNA FISH is available upon request
Data analysis	Details are in the GEO submission

For manuscripts utilizing custom algorithms or software that are central to the research but not yet described in published literature, software must be made available to editors/reviewers upon request. We strongly encourage code deposition in a community repository (e.g. GitHub). See the Nature Research guidelines for submitting code & software for further information.

Data

Policy information about availability of data

All manuscripts must include a data availability statement. This statement should provide the following information, where applicable:

- Accession codes, unique identifiers, or web links for publicly available datasets
- A list of figures that have associated raw data
- A description of any restrictions on data availability

All ChIP-seq and RNA-seq datasets were deposited at the NCBI GEO database under the accession number GSE104883.

Field-specific reporting

Please select the best fit for your research. If you are not sure, read the appropriate sections before making your selection.

☒ Life sciences ☐ Behavioural & social sciences ☐ Ecological, evolutionary & environmental sciences

For a reference copy of the document with all sections, see nature.com/authors/policies/ReportingSummary-flat.pdf

Life sciences study design

All studies must disclose on these points even when the disclosure is negative.

Sample size	No power analyses were performed to determine sample size. Sample size was based on past experience (for example to determine the number of embryos used in cell fate assays).
Data exclusions	No data were excluded from the analyses.
Replication	All attempts at replication were successful. The minimum number of replicates was least 3, expect for some experiments such as Western Blots which we have only done twice as the results were clear and the controls clean.
Randomization	Randomisation was not required.
Blinding	No specific methods were used for blinding

Reporting for specific materials, systems and methods

Materials & experimental systems

n/a	Involved in the study	
☒	☐	Unique biological materials
☐	☒	Antibodies
☐	☒	Eukaryotic cell lines
☒	☐	Palaeontology
☐	☒	Animals and other organisms
☒	☐	Human research participants

Methods

n/a	Involved in the study	
☐	☒	ChIP-seq
☐	☒	Flow cytometry
☒	☐	MRI-based neuroimaging

Antibodies

Antibodies used

Description Cat. No. Supplier
E2A Sc-763 Santa Cruz
ETO2 Sc-9739 Santa Cruz
LMO2 Mca2744ga AbD Serotec
RING1B D139-3 MBL
RYBP AB3637 Millipore
SCL Sc-12984 Santa Cruz
cTNT Ab8295 Clone 1C11 Abcam
CD31 AF3628 R&D systems
Alexa488
donkey anti- mouse A21202 Thermofisher
Alexa555
donkey anti- goat
A21432
Thermofisher

mSIN3A Sc-994 Santa Cruz
H2A ab18255 Abcam
H3K27me3 ABE44 Millipore
Goat anti-mouse IgG-HRP Sc-2031 Santa Cruz
Donkey anti-rabbit IgG-HRP Sc-2313 Santa Cruz
Donkey anti-goat IgG-HRP Sc-2020 Santa Cruz
Easyblot anti-goat GTX628547-01 Genetex

Easyblot anti-mouse GTX221667-01 Genetex
Easyblot anti-rabbit GTX221666-01 Genetex
FLK1-PE 12-5821-83 eBioscience
CD140a-APC 17-1401-81 eBioscience
ETO2 Sc-9741 X Santa Cruz
SCL rabbit antiserum - -

Validation	All the antibodies used are described in the literature and/or validation is provided by the suppliers. We show validation of the specificity of each antibody used in western blots in Supplementary Material. We have performed control experiments to ensure specificity of the antibodies used in immuno-fluorescence and FACS assays (such as secondary antibody only, staining cells not expressing the protein of interest).

Eukaryotic cell lines

Policy information about cell lines

Cell line source(s)	Mouse ES cells were obtained from appropriate research groups and referenced
Authentication	The cell lines used were not authenticated
Mycoplasma contamination	All the mES cells used were tested negative for mycoplasma
Commonly misidentified lines (See ICLAC register)	We did not used commonly misidentified lines.

Animals and other organisms

Policy information about studies involving animals; ARRIVE guidelines recommended for reporting animal research

Laboratory animals	All animal work was carried out according to UK Home Office regulations under appropriate project licences. Mice aged 8 weeks to 12 weeks old (C57Bl/6xSv129J) were used in time-mating and the embryos collected at E9.5.
Wild animals	The study did not involve wild animals
Field-collected samples	The study did not involve samples collected from the field

ChIP-seq

Data deposition

☒ Confirm that both raw and final processed data have been deposited in a public database such as GEO.

☒ Confirm that you have deposited or provided access to graph files (e.g. BED files) for the called peaks.

Data access links
May remain private before publication.

https://www.ncbi.nlm.nih.gov/geo/query/acc.cgi?acc=GSE104883

Files in database submission

Fastq.gz:
Delta-Input_S6_L001_R1_001.fastq.gz
Delta-Input_S6_L001_R2_001.fastq.gz
Delta-Input_S6_L002_R1_001.fastq.gz
Delta-Input_S6_L002_R2_001.fastq.gz
Delta-Input_S6_L003_R1_001.fastq.gz
Delta-Input_S6_L003_R2_001.fastq.gz
Delta-Input_S6_L004_R1_001.fastq.gz
Delta-Input_S6_L004_R2_001.fastq.gz
ETO2-d4-chip3_S3_L001_R1_001.fastq.gz
ETO2-d4-chip3_S3_L001_R2_001.fastq.gz
ETO2-d4-chip3_S3_L002_R1_001.fastq.gz
ETO2-d4-chip3_S3_L002_R2_001.fastq.gz
ETO2-d4-chip3_S3_L003_R1_001.fastq.gz
ETO2-d4-chip3_S3_L003_R2_001.fastq.gz
ETO2-d4-chip3_S3_L004_R1_001.fastq.gz
ETO2-d4-chip3_S3_L004_R2_001.fastq.gz
H2K119ub-Delta_S7_L001_R1_001.fastq.gz
H2K119ub-Delta_S7_L001_R2_001.fastq.gz
H2K119ub-Delta_S7_L002_R1_001.fastq.gz
H2K119ub-Delta_S7_L002_R2_001.fastq.gz
H2K119ub-Delta_S7_L003_R1_001.fastq.gz
H2K119ub-Delta_S7_L003_R2_001.fastq.gz
H2K119ub-Delta_S7_L004_R1_001.fastq.gz
H2K119ub-Delta_S7_L004_R2_001.fastq.gz

H2K119ub-WT_S3_L001_R1_001.fastq.gz
H2K119ub-WT_S3_L001_R2_001.fastq.gz
H2K119ub-WT_S3_L002_R1_001.fastq.gz
H2K119ub-WT_S3_L002_R2_001.fastq.gz
H2K119ub-WT_S3_L003_R1_001.fastq.gz
H2K119ub-WT_S3_L003_R2_001.fastq.gz
H2K119ub-WT_S3_L004_R1_001.fastq.gz
H2K119ub-WT_S3_L004_R2_001.fastq.gz
H3K27ac-Delta_S10_L001_R1_001.fastq.gz
H3K27ac-Delta_S10_L001_R2_001.fastq.gz
H3K27ac-Delta_S10_L002_R1_001.fastq.gz
H3K27ac-Delta_S10_L002_R2_001.fastq.gz
H3K27ac-Delta_S10_L003_R1_001.fastq.gz
H3K27ac-Delta_S10_L003_R2_001.fastq.gz
H3K27ac-Delta_S10_L004_R1_001.fastq.gz
H3K27ac-Delta_S10_L004_R2_001.fastq.gz
H3K27ac-WT_S9_L001_R1_001.fastq.gz
H3K27ac-WT_S9_L001_R2_001.fastq.gz
H3K27ac-WT_S9_L002_R1_001.fastq.gz
H3K27ac-WT_S9_L002_R2_001.fastq.gz
H3K27ac-WT_S9_L003_R1_001.fastq.gz
H3K27ac-WT_S9_L003_R2_001.fastq.gz
H3K27ac-WT_S9_L004_R1_001.fastq.gz
H3K27ac-WT_S9_L004_R2_001.fastq.gz
H3K27me3-Delta_S1_L001_R1_001.fastq.gz
H3K27me3-Delta_S1_L001_R2_001.fastq.gz
H3K27me3-Delta_S1_L002_R1_001.fastq.gz
H3K27me3-Delta_S1_L002_R2_001.fastq.gz
H3K27me3-Delta_S1_L003_R1_001.fastq.gz
H3K27me3-Delta_S1_L003_R2_001.fastq.gz
H3K27me3-Delta_S1_L004_R1_001.fastq.gz
H3K27me3-Delta_S1_L004_R2_001.fastq.gz
H3K27me3-WT_S8_L001_R1_001.fastq.gz
H3K27me3-WT_S8_L001_R2_001.fastq.gz
H3K27me3-WT_S8_L002_R1_001.fastq.gz
H3K27me3-WT_S8_L002_R2_001.fastq.gz
H3K27me3-WT_S8_L003_R1_001.fastq.gz
H3K27me3-WT_S8_L003_R2_001.fastq.gz
H3K27me3-WT_S8_L004_R1_001.fastq.gz
H3K27me3-WT_S8_L004_R2_001.fastq.gz
H3K4me3-Delta_S4_L001_R1_001.fastq.gz
H3K4me3-Delta_S4_L001_R2_001.fastq.gz
H3K4me3-Delta_S4_L002_R1_001.fastq.gz
H3K4me3-Delta_S4_L002_R2_001.fastq.gz
H3K4me3-Delta_S4_L003_R1_001.fastq.gz
H3K4me3-Delta_S4_L003_R2_001.fastq.gz
H3K4me3-Delta_S4_L004_R1_001.fastq.gz
H3K4me3-Delta_S4_L004_R2_001.fastq.gz
H3K4me3-WT_S5_L001_R1_001.fastq.gz
H3K4me3-WT_S5_L001_R2_001.fastq.gz
H3K4me3-WT_S5_L002_R1_001.fastq.gz
H3K4me3-WT_S5_L002_R2_001.fastq.gz
H3K4me3-WT_S5_L003_R1_001.fastq.gz
H3K4me3-WT_S5_L003_R2_001.fastq.gz
H3K4me3-WT_S5_L004_R1_001.fastq.gz
H3K4me3-WT_S5_L004_R2_001.fastq.gz
HCWT-ETO2-chip3_S6_L001_R1_001.fastq.gz
HCWT-ETO2-chip3_S6_L001_R2_001.fastq.gz
HCWT-ETO2-chip3_S6_L002_R1_001.fastq.gz
HCWT-ETO2-chip3_S6_L002_R2_001.fastq.gz
HCWT-ETO2-chip3_S6_L003_R1_001.fastq.gz
HCWT-ETO2-chip3_S6_L003_R2_001.fastq.gz
HCWT-ETO2-chip3_S6_L004_R1_001.fastq.gz
HCWT-ETO2-chip3_S6_L004_R2_001.fastq.gz
HCWT-ETO2-input3_S5_L001_R1_001.fastq.gz
HCWT-ETO2-input3_S5_L001_R2_001.fastq.gz
HCWT-ETO2-input3_S5_L002_R1_001.fastq.gz
HCWT-ETO2-input3_S5_L002_R2_001.fastq.gz
HCWT-ETO2-input3_S5_L003_R1_001.fastq.gz
HCWT-ETO2-input3_S5_L003_R2_001.fastq.gz
HCWT-ETO2-input3_S5_L004_R1_001.fastq.gz
HCWT-ETO2-input3_S5_L004_R2_001.fastq.gz
Input-WT_S2_L001_R1_001.fastq.gz
Input-WT_S2_L001_R2_001.fastq.gz
Input-WT_S2_L002_R1_001.fastq.gz

Input-WT_S2_L002_R2_001.fastq.gz
Input-WT_S2_L003_R1_001.fastq.gz
Input-WT_S2_L003_R2_001.fastq.gz
Input-WT_S2_L004_R1_001.fastq.gz
Input-WT_S2_L004_R2_001.fastq.gz
sampleWT-1A_S2_L001_R1_001.fastq.gz
sampleWT-1A_S2_L001_R2_001.fastq.gz
sampleWT-1A_S2_L002_R1_001.fastq.gz
sampleWT-1A_S2_L002_R2_001.fastq.gz
sampleWT-1A_S2_L003_R1_001.fastq.gz
sampleWT-1A_S2_L003_R2_001.fastq.gz
sampleWT-1A_S2_L004_R1_001.fastq.gz
sampleWT-1A_S2_L004_R2_001.fastq.gz
sampleWT-1I_S7_L001_R1_001.fastq.gz
sampleWT-1I_S7_L001_R2_001.fastq.gz
sampleWT-1I_S7_L002_R1_001.fastq.gz
sampleWT-1I_S7_L002_R2_001.fastq.gz
sampleWT-1I_S7_L003_R1_001.fastq.gz
sampleWT-1I_S7_L003_R2_001.fastq.gz
sampleWT-1I_S7_L004_R1_001.fastq.gz
sampleWT-1I_S7_L004_R2_001.fastq.gz
sampleWT-2A_S4_L001_R1_001.fastq.gz
sampleWT-2A_S4_L001_R2_001.fastq.gz
sampleWT-2A_S4_L002_R1_001.fastq.gz
sampleWT-2A_S4_L002_R2_001.fastq.gz
sampleWT-2A_S4_L003_R1_001.fastq.gz
sampleWT-2A_S4_L003_R2_001.fastq.gz
sampleWT-2A_S4_L004_R1_001.fastq.gz
sampleWT-2A_S4_L004_R2_001.fastq.gz
sample-WT-2I_S6_L001_R1_001.fastq.gz
sample-WT-2I_S6_L001_R2_001.fastq.gz
sample-WT-2I_S6_L002_R1_001.fastq.gz
sample-WT-2I_S6_L002_R2_001.fastq.gz
sample-WT-2I_S6_L003_R1_001.fastq.gz
sample-WT-2I_S6_L003_R2_001.fastq.gz
sample-WT-2I_S6_L004_R1_001.fastq.gz
sample-WT-2I_S6_L004_R2_001.fastq.gz
WTCHG_45129_224_1_sequence.txt.gz
WTCHG_45129_224_2_sequence.txt.gz
WTCHG_45129_225_1_sequence.txt.gz
WTCHG_45129_225_2_sequence.txt.gz
WTCHG_45129_227_1_sequence.txt.gz
WTCHG_45129_227_2_sequence.txt.gz
WTCHG_45129_228_1_sequence.txt.gz
WTCHG_45129_228_2_sequence.txt.gz
WTCHG_45130_224_1_sequence.txt.gz
WTCHG_45130_224_2_sequence.txt.gz
WTCHG_45130_225_1_sequence.txt.gz
WTCHG_45130_225_2_sequence.txt.gz
WTCHG_45130_227_1_sequence.txt.gz
WTCHG_45130_227_2_sequence.txt.gz
WTCHG_45130_228_1_sequence.txt.gz
WTCHG_45130_228_2_sequence.txt.gz
WTCHG_45131_262_1_sequence.txt.gz
WTCHG_45131_262_2_sequence.txt.gz
WTCHG_45131_263_1_sequence.txt.gz
WTCHG_45131_263_2_sequence.txt.gz
WTCHG_45131_290_1_sequence.txt.gz
WTCHG_45131_290_2_sequence.txt.gz
WTCHG_45131_291_1_sequence.txt.gz
WTCHG_45131_291_2_sequence.txt.gz
WTCHG_45132_262_1_sequence.txt.gz
WTCHG_45132_262_2_sequence.txt.gz
WTCHG_45132_263_1_sequence.txt.gz
WTCHG_45132_263_2_sequence.txt.gz
WTCHG_45132_290_1_sequence.txt.gz
WTCHG_45132_290_2_sequence.txt.gz
WTCHG_45132_291_1_sequence.txt.gz
WTCHG_45132_291_2_sequence.txt.gz
WTCHG_50373_265_1_sequence.txt.gz
WTCHG_50373_265_2_sequence.txt.gz
WTCHG_50373_266_1_sequence.txt.gz
WTCHG_50373_266_2_sequence.txt.gz
Processed files:
WTH2aubK119_broad_peaks.broadPeak

WTH3K27ac_broad_peaks.broadPeak
WTH3K27me3_broad_peaks.broadPeak
WTH3K4me3_broad_peaks.broadPeak
MutH2K119ub_broad_peaks.broadPeak
MutH3K27ac_broad_peaks.broadPeak
MutH3K27me3_broad_peaks.broadPeak
MutH3K4me3_broad_peaks.broadPeak
sampleWT_1_broad_peaks.broadPeak
sampleWT_2_broad_peaks.broadPeak
merged_RYBP_WT_broadPeak.bed
J1_SCL_optimum_narrowPeak.bed
ETO2_chip3_3206peaks.txt
ETO2_chip3_vs_input3_peaks.narrowPeak
ETO2chip3vsnoinput_270417_peaks.narrowPeak
cpm_table_J1_with_annotation.txt

Excel files: metadataspreadsheet
GEO_ETO2CHIP_hediac.xls
GEO_HistoneChipRx_hediac.xls
GEO_RYBP_CHIP_hediac.xls
GEO_SCL_CHIP_hediac.xls
GEO_SCLRNAseq_hediac.xlsx

Genome browser session
(e.g. UCSC)

No longer applicable

Methodology

Replicates

ETO2_Chip: 2 replicates
ETO2_Chip_replicate1
ETO2_Chip_input1
ETO2_Chip_replicate2
RYBP_Chip: 2 replicates
sampleWT-1A
sampleWT-2A
sampleWT-1I (input1)
sampleWT-2I (input2)
HistoneChipRx: 1 replicate per histone modification
H2K119ub-Delta
H2K119ub-WT
H3K27me3-Delta
H3K27me3-WT
H3K27ac-Delta
H3K27ac-WT
H3K4me3-Delta
H3K4me3-WT
Delta-Input
Input-WT
SCL_Chip: 2 replicates
SCL_Chip_replicate1
SCL_Chip_input1
SCL_Chip_replicate2
SCL_Chip_input2
SCL_RNAseq: 3 replicates
j1.wt1
j1.scl1
j1.wt2
j1.scl2
j1.wt3
j1.scl3

Sequencing depth

ETO2_Chip:
ETO2_Chip_replicate1; 221,532,610 total reads; 144112252 mapped reads; 37bp paired-end
ETO2_Chip_input1: 120,657,840 total reads; 70,978,652 mapped reads; 37bp paired-end
ETO2_Chip_replicate2: 106,431,788 total reads; 70,978,652 mapped reads; 37bp paired-end
RYBP_Chip
sampleWT-1A: 108,571,980 total reads; 68,728,582 mapped reads; 37bp paired-end
sampleWT-2A: 72,460,170 total reads; 45,738,596 mapped reads; 37bp paired-end
sampleWT-1I (input1): 132,057,930 total reads; 91,013,290 mapped reads; 37bp paired-end
sampleWT-2I (input2): 83,958,126 total reads; 51,387,830 mapped reads; 37bp paired-end
HistoneChipRx:

H2K119ub-Delta : 78,257,316 total reads; 56,546,714 mapped reads; 37bp paired-end
H2K119ub-WT: 86,928,868 total reads; 63,864,268 mapped reads; 37bp paired-end
H3K27me3-Delta: 79,443,550 total reads; 30,545,340 mapped reads; 37bp paired-end
H3K27me3-WT: 75,047,812total reads; 32,980,936 mapped reads; 37bp paired-end
H3K27ac-Delta: 74,058,750 total reads; 49,532,274 mapped reads; 37bp paired-end
H3K27ac-WT: 55,559,129 total reads; 55,559,129 mapped reads; 37bp paired-end
H3K4me3-Delta: 89,521,650 total reads; 49,582,330 mapped reads; 37bp paired-end
H3K4me3-WT: 79,162,084 total reads; 43,904,728 mapped reads; 37bp paired-end
Delta-Input: 60,574,700 total reads; 36,528,760 mapped reads; 37bp paired-end
Input-WT: 72,123,014 total reads; 45,388,646 mapped reads; 37bp paired-end

Antibodies

SCL antibody: SCL rabbit antiserum (in house)
RYBP antibody: AB3637 Millipore
ETO2 antibody: Sc-9741 X Santa Cruz
H3K4me3 ab8580 Abcam
H3K27ac ab 4729 Abcam
H3K27me3 39155 Active motif
H2AK119ub D27C4 Cell Signaling
See table in Material and Methods section.

Peak calling parameters

RYBP Chip:
Chip-seq reads were aligned to the mm9 genome using Bowtie v1.1.2b with the following parameters: -q -p 8 --chunkmbs 10000 -n 2 -m 2
Peaks were called using Macs2 v2.0.10 with the following parameters --broad-cutoff 0.1
SCL Chip:
Chip-seq reads were aligned to the mm9 genome using Bowtie v1.1.2 with the following parameters bowtie -q -n 2 -m 2
Peaks were called using Macs2 v2.0.10 with the following parameters -B -q 0.01
The two replicates were merged and peak called using IDR: https://sites.google.com/site/anshulkundaje/projects/idr
ETO2 Chip:
mapping : bowtie -m 1 --maxIns 350 --lanes 4
peak calling were performed using Macs2 v2.0.10 with the following parameters -B -q 0.05 --to-large
Histone ChipRx:
Chipseq reads were aligned to the mm9 and drosophila dm3 genome references.
mapping : bowtie -m 1 --maxIns 350 --lanes 4
broadPeak files were generated using Macs2 v2.0.10 with the following parameters --broad-cutoff 0.05

Data quality

Chipseq reads were aligned to the mm9 genome reference. Data were analysed using in-house pipeline that automatically assesses the quality of data, trims adaptors, merges short reads, maps reads to the reference genome, and filters data for duplicates: http://userweb.molbiol.ox.ac.uk/public/telenius/PipeSite.html

Software

Sequences were de-multiplexed, aligned to the Mus musculus (mm9) genome using Bowtie (version 1.1.2), and filtered to remove duplicate mapped reads.
for un-mapping reads : trim_galore -- length 10, --noFlash
removing duplicates : samtools rmdup
no ploidy region (bedtools pairtobed)
reconstructing sequenced fragments from R1+R2 read pairs (bedtools bedpe | cut -f 1,2,6) - for visualisation
pileup of fragments (bedtools genomecov -counts)
generating bigwig tracks (ucsctools bedGraphToBigWig)
peak calling were performed using Macs2 v2.0.10
Peak calling was performed with MACS2 (version 2.0.10) using default parameters for SCL and ETO2 ChIP; the -- broad option was applied for RYBP ChIP.
MIG (Multi-Image Genome viewer) was used to visualise the data, filter and identify peaks. (McGowan et al, Bioinformatics 29, 2477-2478 (2013)).
Data for heatmaps and meta gene plots were generated using HOMER annotatePeaks command and visualised with Java_Treeview.
GO analysis of genomic regions was performed with Stanford GREAT online tool.
For histoneChipRx: Tag directories were created using Homer and the generated BedGraphs were visualised on UCSC Genome Browser as custom tracks, after scaling the tag densities by the normalisation factor for each histone mark.
Histograms and heatmaps were generated using AnnotatePeaks.pl command with up to -100kb to 100kb around the indicated genomic regions separated into 25 equally sized bin.

Flow Cytometry

Plots

Confirm that:

☒ The axis labels state the marker and fluorochrome used (e.g. CD4-FITC).

☒ The axis scales are clearly visible. Include numbers along axes only for bottom left plot of group (a 'group' is an analysis of identical markers).

☒ All plots are contour plots with outliers or pseudocolor plots.

☒ A numerical value for number of cells or percentage (with statistics) is provided.

Methodology

Sample preparation	Embryoid bodies from mouse embryonic stem cells were dissociated into single cell suspension with trypsin/EDTA 0.25% for 3 min in a water bath.<br>The cardiac cultures were dissociated using 0.25% trypsin/EDTA for 5 mins at 37˚C to quickly lift the monolayer cells off the plate, then a single cell suspension was achieved with a solution of collagenase/DNase (PBS with 20% serum, 10 mg/ml collagenase A, 10 mg/ml collagenase B and 10 microg/ml DNase I 5 to 10 mins at 37˚C.
Instrument	BD LSR Fortessa X20 fitted with 4 lasers (405,488,561, 640nm).
Software	BD FACSDIVA SOFTWARE
Cell population abundance	Samples were sorted on FACS Aria II (BD). After the sort, the sorted samples were re-run to check that they fall within the sorting gate.<br>Supplementary Figure 3 shows representative post-sort purity examples.
Gating strategy	Gating strategy is shown in supplementary Figure 3. First a morphology gate (FSC versus SSC) is drawn. Then this morphology gate is reported to a 2nd window (FCS versus pulse width) to define the singulets and exclude the doublets. The singulet gate is reported to a 3rd window (FSC versus live/dead stain) to exclude the dead cells. The intersection of the morphology, singulet and live gates are then reported to all the downstream windows displaying fluorescence X and Y parameters.<br>For SCL intra-cellular FACS: SCL null cells are used to set up the SCL positive gate in the samples.<br>For mcherry positive cells: we used ES cells non expressing mcherry to set up the mcherry positive gate.<br>Positive and negative population are defined using single stain and FMO (Fluorescence minus one) tubes.

☒ Tick this box to confirm that a figure exemplifying the gating strategy is provided in the Supplementary Information.
